# The role of DNA (de)methylation in immune responsiveness of Arabidopsis

**DOI:** 10.1111/tpj.13252

**Published:** 2016-09-07

**Authors:** Ana López Sánchez, Joost H.M. Stassen, Leonardo Furci, Lisa M. Smith, Jurriaan Ton

**Affiliations:** ^1^ P3 Institute for Translational Plant and Soil BiologyDepartment of Animal and Plant SciencesThe University of SheffieldSheffieldUK

**Keywords:** DNA methylation, defence priming, basal resistance, systemic acquired resistance, transgenerational acquired resistance, *Arabidopsis thaliana*, *Hyaloperonospora arabidopsidis*, E‐MTAB‐3963

## Abstract

DNA methylation is antagonistically controlled by DNA methyltransferases and DNA demethylases. The level of DNA methylation controls plant gene expression on a global level. We have examined impacts of global changes in DNA methylation on the Arabidopsis immune system. A range of hypo‐methylated mutants displayed enhanced resistance to the biotrophic pathogen *Hyaloperonospora arabidopsidis* (*Hpa*), whereas two hyper‐methylated mutants were more susceptible to this pathogen. Subsequent characterization of the hypo‐methylated *nrpe1* mutant, which is impaired in RNA‐directed DNA methylation, and the hyper‐methylated *ros1* mutant, which is affected in DNA demethylation, revealed that their opposite resistance phenotypes are associated with changes in cell wall defence and salicylic acid (SA)‐dependent gene expression. Against infection by the necrotrophic pathogen *Plectosphaerella cucumerina*,* nrpe1* showed enhanced susceptibility, which was associated with repressed sensitivity of jasmonic acid (JA)‐inducible gene expression. Conversely, *ros1* displayed enhanced resistance to necrotrophic pathogens, which was not associated with increased responsiveness of JA‐inducible gene expression. Although *nrpe1* and *ros1* were unaffected in systemic acquired resistance to *Hpa*, they failed to develop transgenerational acquired resistance against this pathogen. Global transcriptome analysis of *nrpe1* and *ros1* at multiple time‐points after *Hpa* infection revealed that 49% of the pathogenesis‐related transcriptome is influenced by NRPE1‐ and ROS1‐controlled DNA methylation. Of the 166 defence‐related genes displaying augmented induction in *nrpe1* and repressed induction in *ros1*, only 25 genes were associated with a nearby transposable element and NRPE1‐ and/or ROS1‐controlled DNA methylation. Accordingly, we propose that the majority of NRPE1‐ and ROS1‐dependent defence genes are regulated in *trans* by DNA methylation.

## Significance Statement

DNA methylation has been implicated in defence priming, but it is not fully understood what types of plant immunity are influenced or to what extent DNA methylation is involved. We addressed these questions by comprehensive phenotypic and transcriptomic analysis of Arabidopsis mutants that are oppositely affected in DNA methylation. We show that DNA (de)methylation processes play critical roles in certain types of innate, and demonstrate acquired immunity and that DNA (de)methylation exerts a global influence on the responsiveness of the defence‐related transcriptome, predominantly via *trans*‐regulatory mechanisms.

## Introduction

Plants activate defence mechanisms in response to microbial attack. This innate immune response operates through conserved signalling mechanisms, such as the recognition of microbe‐ or damage‐associated molecular patterns (MAMPs and DAMPs), production of reactive oxygen and nitrogen species, and induction of plant defence hormones, such as salicylic acid (SA) and jasmonic acid (JA; Thomma *et al*., 2001). Together, these signalling events lead to a coordinated transcriptional response that controls production of long‐distance defence signals, pathogenesis‐related proteins and antimicrobial metabolites. Expression of innate immunity is often transient, but can lead to a form of acquired immunity that manifests itself as a ‘priming’ of inducible defences (Prime‐A‐Plant Group *et al*., 2006).

Primed plants respond faster and stronger to a secondary defence stimulus, such as pathogen attack, wounding, or treatment with chemical defence elicitors (Conrath, 2006; Frost *et al*., 2008; Ahmad *et al*., 2010). Plants can develop different types of defence priming, which are controlled by partially different signalling mechanisms. Some priming responses are triggered by plant–microbe interactions, such as pathogen‐induced systemic acquired resistance (SAR; Durrant and Dong, 2004) or root microbe‐induced systemic resistance (Van Wees *et al*., 2008), whereas others can be induced by application of specific chemicals, such as beta‐amino butyric acid (BABA; Luna *et al*., 2014a). On a temporal scale, there are types of defence priming that are relatively short‐lived and disappear over days (Luna *et al*., 2014b), whereas priming of SA‐ and JA‐dependent defences are long‐lasting (Worrall *et al*., 2012; Luna *et al*., 2014b), and can even be transmitted to the next generation, resulting in transgenerational acquired resistance (TAR; Luna *et al*., 2012; Rasmann *et al*., 2012; Slaughter *et al*., 2012). The durable and heritable character of priming of SA‐dependent immunity have suggested involvement of epigenetic regulatory mechanisms, such as chromatin remodelling and DNA (de)methylation, which can account for long‐lasting changes in defence gene responsiveness (Jaskiewicz *et al*., 2011; Pastor *et al*., 2013; Conrath *et al*., 2015).

DNA methylation is critical for diverse biological processes including gene expression and genome stability. The pattern of DNA methylation is controlled by an equilibrium between methylation and demethylation activities (Law and Jacobsen, 2010). In plants, cytosine‐specific DNA methyltransferases (MTases) are responsible for DNA methylation, which add a methyl group to the fifth carbon of cytosines (Pavlopoulou and Kossida, 2007). *De novo* DNA methylation is controlled by small interfering RNAs (siRNAs). This RNA‐directed DNA methylation (RdDM) is mediated by two overlapping pathways, controlling initiation and establishment of DNA methylation in every sequence context (CG, CHG and CHH; H = any nucleotide but G; Matzke and Mosher, 2014). Initiation of *de novo* DNA methylation involves transcription of target sequences by DNA‐DEPENDENT RNA POLYMERASE II (Pol II). Some Pol II transcripts can be amplified by RNA‐DEPENDENT RNA POLYMERASE 6 (RDR6), which are processed by DICER‐LIKE (DCL) 2 and 4 into 21‐22 nucleotide (nt) siRNAs. These siRNAs can induce low levels of DNA methylation via DNA‐DEPENDENT RNA POLYMERASE V (Pol V) and the DNA methyltransferase DOMAINS REARRANGED METHYLTRANSFERASE 2 (DRM2; Nuthikattu *et al*., 2013). This initiation of DNA methylation activates the second RdDM pathway, in which DNA‐DEPENDENT RNA POLYMERASE IV (Pol IV) generates single‐stranded RNA molecules, which are copied and amplified into double‐stranded RNAs by RNA‐DEPENDENT RNA POLYMERASE 2 (RDR2), processed into 24 nt siRNAs by DCL3, and loaded onto ARGONAUTE 4 (AGO4). The latter protein enables base pairing between the siRNA with Pol V‐produced RNA transcripts, after which DRM2 is recruited for establishment of DNA methylation (Matzke and Mosher, 2014). DRM2‐dependent CHH methylation cannot be maintained in the absence of siRNAs, and requires on‐going activity by the Pol IV‐RDR2‐dependent RdDM pathway (Law and Jacobsen, 2010). However, once established, asymmetrical CHH methylation can spread into symmetrical CG or CHG methylation that is stably preserved through DNA replication by METHYLTRANSFERASE 1 (MET1) and CHROMOMETHYLASE 3 (CMT3), respectively. DNA demethylation in plants occurs either passively, during DNA replication, or can occur actively through DNA glycosylase/lyase activity (Zhu, 2009). In Arabidopsis, four DNA glycosylases/lyases have been identified: REPRESSOR OF SILENCING 1 (ROS1), DEMETER (DME), DEMETER‐LIKE 2 (DML2) and DEMETER‐LIKE 3 (DML3), where ROS1 is predominantly responsible for DNA demethylation in vegetative tissues (Penterman *et al*., 2007; Zhu, 2009; Gong and Zhu, 2011).

Recently, DNA methylation and chromatin modifications have emerged as a potential regulatory mechanism of defence priming. Arabidopsis mutants impeded in DNA methylation have been reported to show increased basal resistance to (hemi)biotrophic pathogens (López *et al*., 2011; Dowen *et al*., 2012; Luna *et al*., 2012; Yu *et al*., 2013). Specifically, mutants in non‐CG methylation, such as the Pol IV/Pol V mutant *nrpd2*, the Pol V mutant *nrpe1* and the MTase triple mutant *ddm1 ddm2 cmt3*, display constitutive priming of SA‐dependent *PR1* gene expression (López *et al*., 2011; Luna *et al*., 2012). Other studies have shown that infection of Arabidopsis by the hemi‐biotrophic pathogen *P. syringae* pv. *tomato* DC3000 (*Pst* DC3000) reduces DNA methylation (Pavet *et al*., 2006; Dowen *et al*., 2012; Yu *et al*., 2013), offering a plausible explanation for long‐term and transgenerational defence gene priming upon enduring disease stress. However, despite evidence for *cis*‐regulation of defence gene priming by histone modifications (Jaskiewicz *et al*., 2011; López *et al*., 2011; Luna *et al*., 2012), the relationship between DNA demethylation and defence gene priming is less well documented. In a pioneering study, Dowen *et al*. (2012) reported a correlation between pathogen‐induced DNA hypo‐methylation and pathogen‐induced transcription of proximal genes, suggesting that reduced DNA methylation contributes to regulation of pathogen‐induced gene expression. However, it remained unclear in how far pathogen‐induced DNA hypo‐methylation contributes to transcriptional priming of defence genes. Mutants defective in DNA methylation show constitutive priming of *PR1* gene expression (López *et al*., 2011; Luna *et al*., 2012), demonstrating that DNA hypo‐methylation primes *PR1* gene induction. Interestingly, however, the promoter of *PR1* is normally not methylated. Furthermore, Slaughter *et al*. (2012) found that transgenerational priming of the *PR1* gene in isogenic progeny from BABA‐treated plants is not associated with changes in DNA methylation of *PR1*. Together, these results suggest that regulation of defence gene priming by DNA methylation is not solely based on *cis*‐acting mechanisms.

To date, the exact mechanisms by which DNA methylation controls plant immunity remains unclear. Further investigation is required to establish what types of plant immunity are influenced by DNA methylation, which regulatory mechanisms of DNA (de)methylation control plant immunity, and how DNA methylation regulates defence gene priming on a genome‐wide scale. Here, we have addressed these questions through comprehensive phenotypic and transcriptomic analysis of Arabidopsis mutants that are oppositely affected in DNA methylation, but that do not express developmental growth phenotypes. Our study reveals that DNA (de)methylation processes play critical roles in certain types of innate and acquired immunity. We furthermore show that DNA (de)methylation exerts a global influence on the responsiveness of the defence‐related transcriptome via predominantly *trans*‐regulatory mechanisms.

## Results

### Opposite effects of DNA methylation and DNA demethylation on basal resistance to *Hyaloperonospora arabidopsidis*

**Figure 1:**
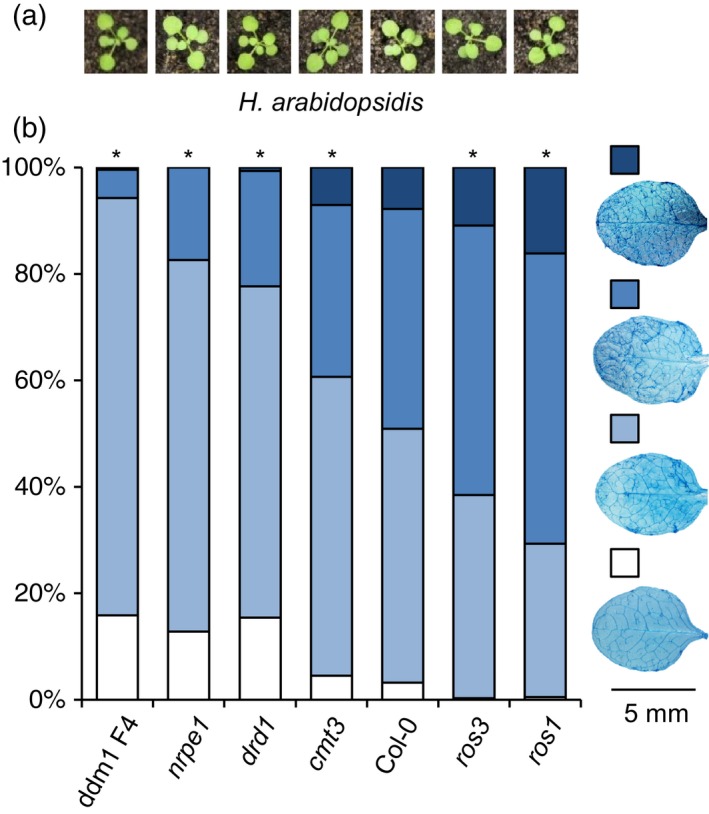
Basal resistance to *Hyaloperonospora arabidopsidis* in *Arabidopsis thaliana* mutants that are affected in DNA (de)methylation. (a) Growth phenotypes of tested Arabidopsis genotypes before infection. Genotypes correspond to those of the bars in (b) below each picture. (b) Levels of basal resistance to *H. arabidopsidis* (*Hpa*) in DNA methylation mutants (*ddm1* F4, *nrpe1*,* drd1*, and *cmt3*) and DNA demethylation mutants (*ros3* and *ros1*). Six days after spray inoculation of 3‐week‐old plants (10^5^ conidiospores ml^−1^), 200 leaves from 35 plants per genotype were microscopically assigned to different *Hpa* colonization classes following trypan blue staining. Shown are relative numbers of leaves assigned to different colonization classes. Inserts show representative levels of classes. Asterisks indicate statistically significant differences in class distributions compared to Col‐0 (χ^2^ test; *P *<* *0.05).

To determine impacts of DNA (de)methylation on resistance against biotrophic pathogens, we evaluated a range of Arabidopsis mutants in DNA (de)methylation mechanisms for basal resistance to the obligate biotrophic oomycete *Hyaloperonospora arabidopsidis* (*Hpa*). To prevent pleiotropic effects of developmental phenotypes, we only selected mutants with normal (wild‐type) growth phenotypes under the conditions of our patho‐assays (Figure 1a). T‐DNA insertions in *ros1* (SALK_135293), *ros3* (SALK_022363C) and *cmt3* (SALK_148381) were confirmed by PCR of genomic DNA (Figure S1a), while transcriptional knock‐down of *ROS1* and *NRPE1* gene expression was confirmed by reverse‐transcriptase quantitative PCR (RT‐qPCR) analysis in *ros1* and *nrpe1*, respectively (Figure S1b). Three‐week‐old seedlings were spray‐inoculated with *Hpa* conidiospores and collected 6 days later for trypan blue staining. Microscopic examination of *Hpa* colonization revealed that two mutants defective in RdDM, *nrpe1* (Pontier *et al*., 2005) and *drd1* (Kanno *et al*., 2004), showed a statistically significant reduction in the number of leaves producing conidiospores and oospores (class III and IV; Figure 1b). The *cmt3* mutant, which is defective in maintenance of CHG methylation (Lindroth *et al*., 2001), also showed enhanced resistance in comparison to Col‐0, although to a lesser extent than *nrpe1* and *drd1* (Figure 1b). The *ddm1* mutant, which is affected DNA methylation at all sequence contexts in intergenic regions (Vongs *et al*., 1993; Jeddeloh *et al*., 1998; Zemach *et al*., 2013), was tested in the fourth generation of homozygosity and showed the strongest level of resistance amongst all genotypes tested (Figure 1b). In contrast to the hypo‐methylated mutants, the DNA glycosylase mutant *ros1*, which is hyper‐methylated at all DNA sequence contexts (Gong *et al*., 2002; Zhu *et al*., 2007), was significantly more susceptible to *Hpa* than Col‐0 plants (Figure 1b). This enhanced susceptibility was similar to that of SA‐insensitive *npr1* plants (Cao *et al*., 1994; Figure S2a). The *ros3* mutant, which is affected in an RNA‐binding protein that interacts with ROS1 (Zheng *et al*., 2008), also showed enhanced susceptibility to *Hpa* (Figure 1b), although this phenotype was not consistent over multiple experiments (Figure S2a). Conversely, all other mutants tested showed similar resistance phenotypes between independent experiments (Figure S2a). Together, these results point to opposite roles of DNA methylation and DNA demethylation in basal resistance to *Hpa*. Subsequent experiments focused on the hypo‐methylated *nrpe1* mutant and hyper‐methylated *ros1* mutant, whose *Hpa* resistance phenotypes were confirmed by qPCR quantification of oomycete biomass (Figure S2b).

### DNA methylation regulates effectiveness of callose deposition and SA‐dependent *PR1* gene induction upon *Hpa* infection

**Figure 2:**
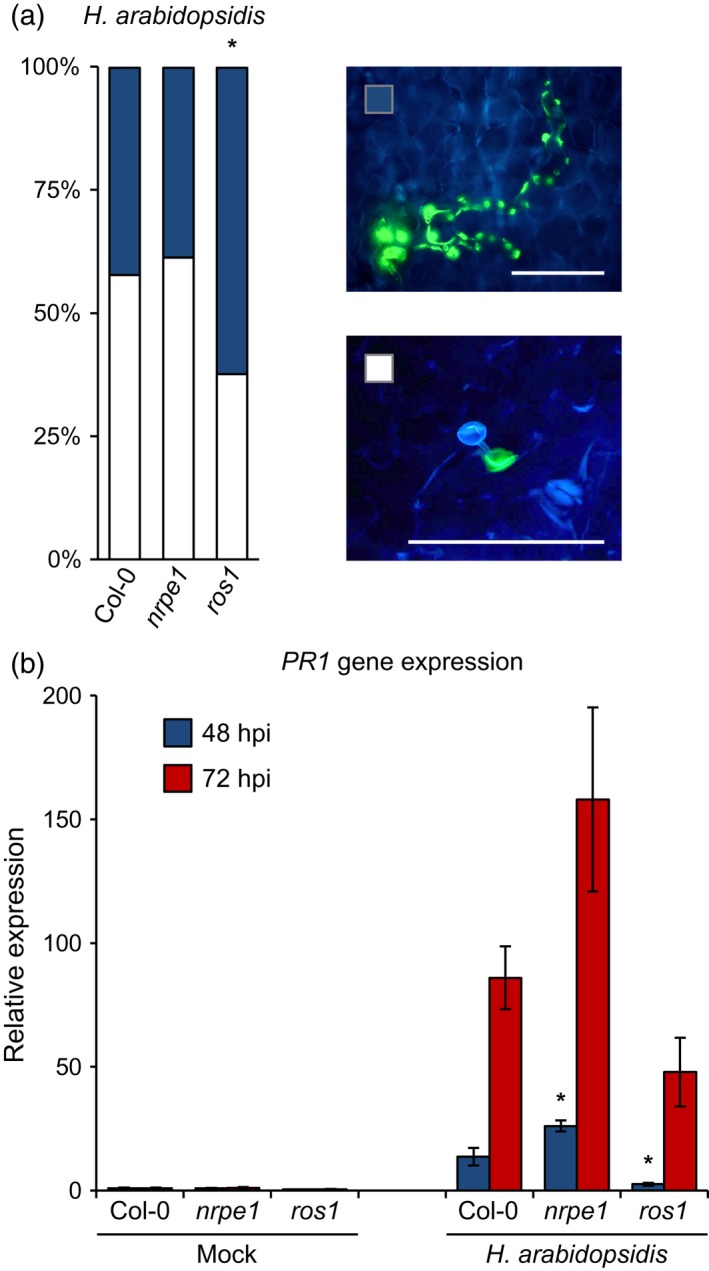
Effectiveness and responsiveness of inducible defences against *H. arabidopsidis* in *nrpe1*,* ros1* and Col‐0. (a) Effectiveness of callose deposition against *Hpa* infection at 48 h after inoculation of 3‐week‐old plants (10^5^ conidiospores ml^−1^). Defence phenotypes were determined by epifluorescence microscopy in at least 10 leaves per genotype, and assigned to two different classes based on presence or absence of successful penetration into the mesophyll by *Hpa*. Inserts on the right show an example of each class. Germinating *Hpa* spores appear in blue (calcofluor white‐stained) and callose deposition is indicated by the presence of yellow staining (analine blue‐stained). Asterisks indicate statistically significant differences in class distributions compared to Col‐0 (χ^2^ test; *P *<* *0.05). Scale bars = 100 μm. (b) RT‐qPCR quantification of *PR1* gene expression in Col‐0, *nrpe1* and *ros1* at 48 and 72 h after inoculation with *Hpa* or mock treatment. Data represent mean values of relative expression (±SEM) from four biologically replicated samples. Asterisks indicate statistically significant differences in comparison to Col‐0 (Student's *t*‐test; *P *<* *0.05).

Reinforcement of the cell wall by deposition of callose‐rich papillae contributes to slowing down pathogen colonization at relatively early stages of infection (Luna *et al*., 2011; Ellinger *et al*., 2013; Voigt, 2014). To determine the role of DNA (de)methylation in this induced defence layer against *Hpa*, we compared the effectiveness of callose deposition in relation to *Hpa* colonization between the wild‐type Col‐0, hypo‐methylated *nrpe1*, and hyper‐methylated *ros1*. To this end, leaves were collected at 48 h post inoculation (hpi) for calcofluor/analine blue double staining and analysed by epifluorescence microscopy. To assess the defence‐contributing activity of callose, all germinating spores were assigned to two mutually exclusive classes: (i) spores that were effectively arrested by callose and (ii) spores that were not arrested by callose. Using this classification, the *ros1* mutant showed a statistically significant reduction in callose effectiveness in comparison to Col‐0 plants (χ^2^; *P *<* *0.001; Figure 2a). This indicates that the enhanced DNA methylation in this mutant represses the effectiveness of callose deposition.

In addition to cell wall defence, resistance to *Hpa* relies on post‐invasive SA‐dependent defences (Lawton *et al*., 1995; Thomma *et al*., 1998; Ton *et al*., 2002). To examine whether DNA (de)methylation affects SA‐dependent defences, we quantified relative transcript accumulation of the SA‐inducible *PR1* marker gene at 48 and 72 hpi with *Hpa*, using RT‐qPCR (Figure 2b). Consistent with previous results (López *et al*., 2011), the more resistant *nrpe1* mutant displayed a stronger induction of the *PR1* gene, which was statistically significant at 48 hpi with *Hpa* (*P* = 0.026). Conversely, the more susceptible *ros1* mutant showed repressed *PR1* induction at 48 hpi compared to Col‐0 (*P* = 0.028). As the *nrpe1* mutant does not show constitutive expression of *PR1* gene, we conclude that the DNA hypo‐methylation in *nrpe1* primes SA‐dependent defence against *Hpa*, whereas DNA hyper‐methylation in *ros1* represses this type of defence.

### Role of NRPE1‐ and ROS1‐dependent DNA methylation in basal resistance against necrotrophic fungi

**Figure 3:**
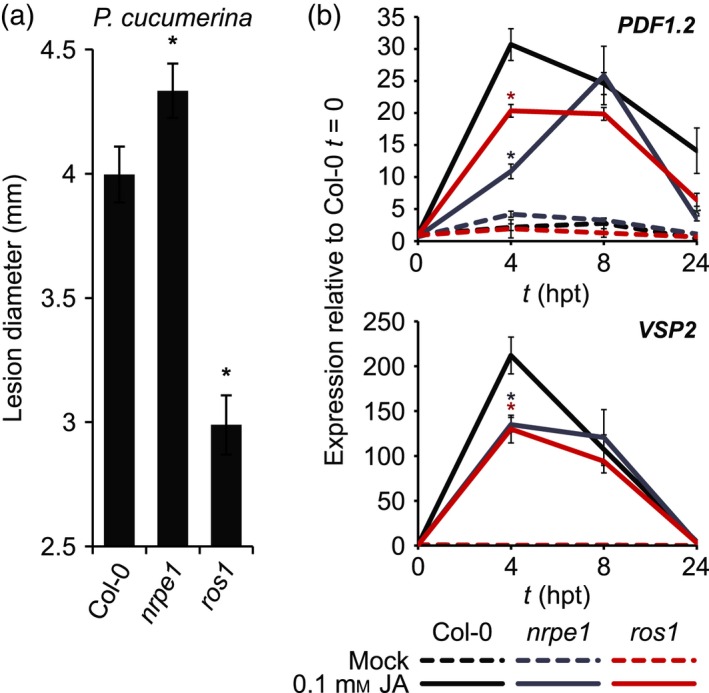
Basal resistance to *Plectosphaerella cucumerina* and JA‐induced gene expression in *nrpe1*,* ros1* and Col‐0. (a) Levels of basal resistance to *P. cucumerina*. Shown are mean lesion diameters (±SEM; 27 plants) at 6 days after droplet inoculation of 4.5‐week‐old plants. Asterisks indicate statistically significant differences between Col‐0 and mutant plants (Student's *t*‐test; *P *<* *0.05). (b) RT‐qPCR quantification of *PDF1.2* and *VSP2* gene expression in Col‐0, *nrpe1* and *ros1* at 0, 4, 8 and 24 h after spraying with 0.1 mm jasmonic acid (JA). Data represent mean values of relative expression (±SEM; *n* = 3). Asterisks indicate statistically significant differences in comparison to Col‐0 samples (Student's *t*‐test; *P* < 0.05).

López *et al*. (2011) demonstrated that mutants in RdDM display enhanced susceptibility to the necrotrophic fungus *Plectosphaerella cucumerina*, which is associated with repressed responsiveness of JA‐dependent defence genes. To examine whether the increased level of DNA methylation in *ros1* has an opposite effect on basal resistance to necrotrophic fungi, we compared 4.5‐week Col‐0, *nrpe1* and *ros1* for basal resistance against the Ascomycete fungus *P. cucumerina*. Basal resistance was quantified by necrotic lesion diameter, which is a reliable parameter to assess necrotrophic colonization by this fungus after droplet inoculation (Ton and Mauch‐Mani, 2004; Pétriacq *et al*., 2016). At 6 days post inoculation, the *nrpe1* mutant developed larger lesions than Col‐0 (Figures 3a and S3a), confirming previous results by López *et al*. (2011). Conversely, *ros1* plants displayed significantly smaller necrotic lesions than Col‐0 (Figures 3a and S3a), indicating enhanced basal resistance to *P. cucumerina*. The disease phenotypes of *nrpe1* and *ros1* were validated by qPCR quantification of fungal DNA (Figure S3b), confirming that both mutants are oppositely affected in disease resistance to *P. cucumerina*. Furthermore, similar results were obtained by quantifying microscopic colonization by a different necrotrophic fungus, *A. brassicicola* (Figure S3c). It can thus be concluded that DNA hyper‐methylation in the *ros1* mutant boosts basal disease resistance to necrotrophic fungi.

Basal resistance against *P. cucumerina* and *A. brassicicola* partially relies on JA‐dependent defences (Thomma *et al*., 1998, 1999; Ton and Mauch‐Mani, 2004). To investigate whether the enhanced resistance of *ros1* is based on increased sensitivity of JA‐inducible defence gene expression, we analysed plants for *PDF1.2* and *VSP2* expression at 0, 4, 8 and 24 h after spraying of the leaves with 50 mm JA. Consistent with the earlier notion that mutations in RdDM repress defence gene responsiveness to JA (López *et al*., 2011), the *nrpe1* mutant showed significantly lower and/or delayed JA induction of both genes in comparison to wild‐type plants (Figure 3b). Surprisingly, despite the fact that the *ros1* mutant was more resistant to both *P. cucumerina* and *A. brassicicola* (Figures 3a and S3), it also showed repressed induction of *PDF1.2* and *VSP2* by JA, which was statistically significant at 4 h post treatment with JA (Figure 3b). Thus, increased resistance of *ros1* to necrotrophic fungi is not based on primed responsiveness of JA‐inducible gene expression.

### ROS1‐dependent demethylation does not play a role in within‐generation SAR, but is required for TAR

**Figure 4:**
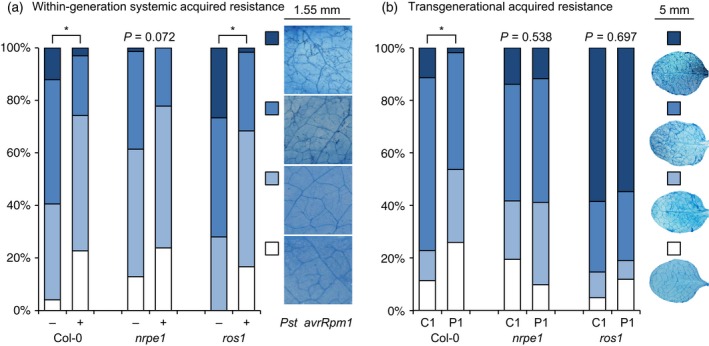
Systemic acquired resistance (SAR) and transgenerational acquired resistance (TAR) in Col‐0, *nrpe1* and *ros1*. (a) Quantification of within‐generation SAR against *Hpa*. Four leaves of 4.5‐week‐old plants were infiltrated with either avirulent *Pseudomonas syringae* pv. *tomato* DC3000 *avrRpm1* (*Pst avrRpm1*) or 10 mm MgSO_4_ (mock). Three days after SAR induction, plants were spray inoculated with *Hpa* (10^5^ conidiospores ml^−1^). At 6 days after inoculation, 4–6 leaves from 15 plants per genotype were stained with trypan blue and microscopically assigned to different *Hpa* colonization classes (right panels). Asterisks indicate statistically significant differences in class distributions between SAR‐ and mock‐treated plants (χ^2^ test; *P* < 0.05). (b) Quantification of TAR against *Hpa* in P1 and C1 progenies from *Pst* DC3000‐ and mock‐inoculated plants, respectively. Parental plants were spray‐inoculated three consecutive times at 3–4 day intervals with *Pst* DC3000 or 10 mm MgSO_4_ (mock), and allowed to set seed. Leaves of 3‐week‐old progenies were inoculated with *Hpa* (10^5^ conidiospores ml^−1^) and examined for pathogen colonization 6 days later, as detailed in the legend of Figure 1(a). Asterisks indicate statistically significant differences in class distributions between P1 and C1 progenies (χ^2^ test; *P *<* *0.05).

Systemic acquired resistance is a pathogen‐inducible form of acquired immunity that is expressed systemically (Durrant and Dong, 2004). Recently, it was shown that pathogen‐induced acquired immunity can be transmitted to following generations in Arabidopsis (TAR; Slaughter *et al*., 2012; Luna *et al*., 2012). This resistance could be mimicked by genetic mutations in the DNA methylation machinery (Luna and Ton, 2012; Luna *et al*., 2012), suggesting that DNA demethylation is responsible for the generation and/or transmission of the response. To investigate the role of NRPE1‐ and ROS1‐dependent DNA (de)methylation during within‐generation SAR, three lower leaves of 4.5‐week‐old plants were infiltrated with avirulent *Pseudomonas syringae* pv. *tomato* DC3000 carrying the avirulence gene *avrRpm1 (Pst avrRpm1)*. Three days after SAR induction, systemic leaves were challenged with *Hpa*. As expected, SAR‐treated Col‐0 plants displayed a statistically significant reduction in *Hpa* colonization compared to control‐treated plants (Figure 4a). SAR in *Pst avrRpm1*‐infected *nrpe1* plants was borderline statistically significant (*P *=* *0.072), probably due to the masking effect of this mutant's elevated basal resistance (Figure 1a). Notably, the *ros1* mutant was fully capable of mounting a statistically significant SAR response against *Hpa* infection, indicating that ROS1‐dependent DNA demethylation does not play a role in within‐generation SAR.

We then investigated the role of NRPE1‐ and ROS1‐dependent DNA (de)methylation in TAR. To this end, Col‐0, *nrpe1* and *ros1* were inoculated three times with increasing doses of virulent *Pseudomonas syringae pv. tomato* DC3000 (*Pst* DC3000) and allowed to set seed. Three‐week‐old F1 seedlings from *Pst* DC3000‐ (P1) and mock‐treated (C1) parent plants were tested for resistance against *Hpa* (Figure 4b). P1 progeny from *Pst* DC3000‐infected Col‐0 showed increased basal resistance in comparison to C1 progeny from mock‐treated Col‐0 (*P* = 0.017). By contrast, there was no statistically significant difference in *Hpa* resistance between P1 and C1 progenies of *nrpe1* (*P* = 0.538). Levels of resistance in C1 progeny from *nrpe1* were statistically similar to that of P1 progeny from Col‐0 (*P* = 0.148), which is consistent with the notion that reduced DNA methylation mimics TAR (Luna and Ton, 2012; Luna *et al*., 2012). Like the *nrpe1* mutant, P1 and C1 progenies from *ros1* did not show a difference in *Hpa* resistance (*P* = 0.697). However, C1 progeny from *ros1* displayed enhanced susceptibility in comparison to both P1 and C1 progeny of Col‐0 (*P* < 0.001), indicating that the lack of TAR in *ros1* is due to this mutant's inability to transmit and/or express transgenerational acquired immunity.

### NRPE1‐ and ROS1‐dependent DNA (de‐)methylation influences nearly half of the pathogenesis‐related transcriptome

**Figure 5:**
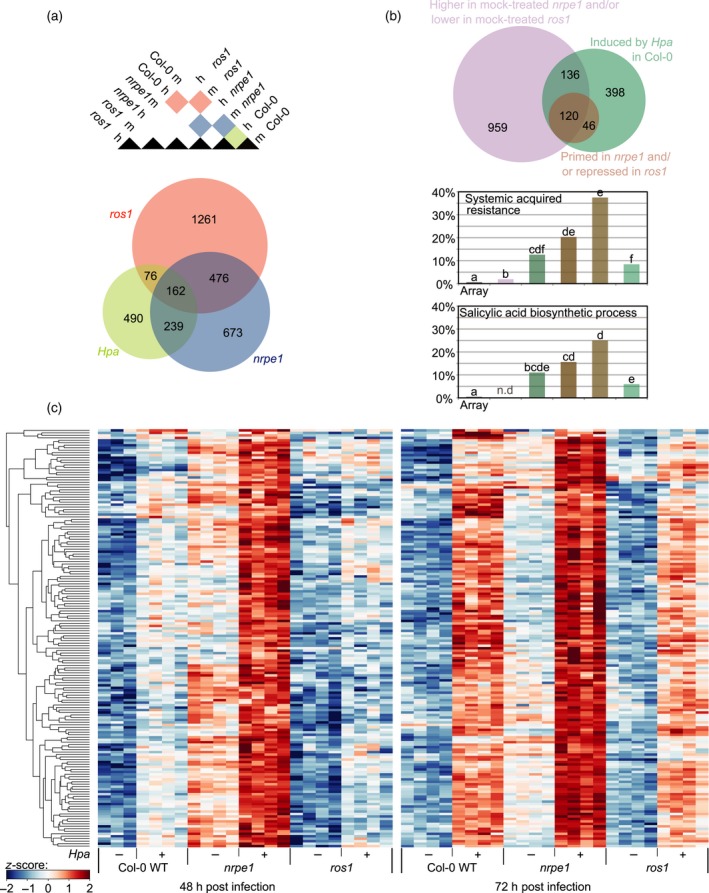
The pathogenesis‐related transcriptome of Col‐0, *nrpe1* and *ros1* during infection by *H. arabidopsidis*. (a) Venn diagram showing numbers of differentially expressed genes at 48 and/or 72 h post inoculation (hpi) between mock‐ (m) and *Hpa*‐inoculated (h) Col‐0 (*Hpa*; green), between Col‐0 and *nrpe1* for any time‐point and condition (*nrpe1*; blue), and between Col‐0 and *ros1* for any time‐point and any condition (*ros1*; red). Each time‐point (48 and 72 hpi) was analysed separately; numbers represent the sum of differentially expressed genes at one or both time‐points. Genes were considered to be differentially expressed at LIMMA‐reported *q*‐value ≤0.01 (global adjust, FDR). (b) *Hpa*‐inducible genes that show augmented induction in *nrpe1* and/or repressed induction in *ros1* are enriched with gene ontology (GO) terms ‘Systemic Acquired Resistance’ (GO: 0009627) and ‘Salicylic Acid Biosynthetic Process’ (GO: 0009697). (c) Transcript levels of all 166 *Hpa‐*inducible genes with augmented induction in *nrpe1* and/or repressed induction in *ros1*. Genes were selected when differentially expressed between *ros1* and *nrpe1*, as well as between Col‐0 and *ros1*, and/or between Col‐0 and *nrpe1*, at either time‐point after inoculation. Heat map projections represent *z*‐scores of transcript levels.

DNA methylation patterns are known to affect gene expression (Law and Jacobsen, 2010). Since *nrpe1* and *ros1* are antagonistically affected in both DNA methylation and responsiveness of *PR1* expression during *Hpa* infection (Figure 2b), we further explored global impacts of both mutations on the pathogenesis‐related transcriptome of *Hpa*‐infected Arabidopsis, using Affymetrix Gene 1.0 ST arrays. To account for transcriptomic responses during expression of penetration defence (48 hpi) and post‐invasive defence during hyphal colonization (72 hpi), we isolated RNA from Col‐0, *nrpe1* and *ros1* at 48 and 72 hpi, respectively. First, we assessed the global impacts of mutations in *NRPE1* and *ROS1* by determining the number of differentially expressed genes between each mutant and Col‐0 at any time‐point and condition (*q *≤* *0.01). This analysis revealed that 1975 and 1150 genes are differentially expressed in the *ros1* and *nrpe1*, respectively. By comparing these gene sets with the 967 genes that are differentially expressed in Col‐0 between mock and *Hpa*‐inoculated leaf samples (i.e. the *Hpa*‐responsive genes), we found that 49% of all *Hpa*‐responsive genes are affected by mutations in *NRPE1* and/or *ROS1* (477/967 = 49%; Figure 5a). Hence, nearly half of the pathogenesis‐related transcriptome of Arabidopsis is controlled directly or indirectly by NRPE1‐ and ROS1‐dependent DNA (de)methylation.

### Defence‐related genes that are primed by DNA hypo‐methylation and/or repressed by DNA hyper‐methylation are strongly enriched with SA‐dependent defence genes

The resistance phenotypes of *nrpe1* and *ros1* to *Hpa* can be caused by constant changes in defence gene expression, changes in defence gene responsiveness to pathogen attack, or a combination of both. Comparison of mock‐inoculated *nrpe1* and *ros1* relative to Col‐0 identified 1215 genes with enhanced expression in *nrpe1* and/or repressed expression in *ros1* at 48 and/or 72 hpi (Figure 5b). Of these, 256 genes were also *Hpa*‐inducible in Col‐0 plants (Figure 5b). We then searched for defence‐related genes with increased *Hpa* responsiveness in the more resistant *nrpe1* mutant (i.e. ‘primed’) and/or repressed responsiveness in the more susceptible *ros1* mutant. To this end, the group of 700 *Hpa*‐inducible genes (shown in green; Figure 5b) were filtered: (i) for a statistically significant difference between *Hpa*‐inoculated *nrpe1* and *ros1* (48 and/or 72 hpi; *q* ≤ 0.01); and (ii) for a statistically significant difference between at least one of the *Hpa*‐inoculated mutants and *Hpa*‐inoculated Col‐0 (48 and/or 72 hpi; *q *≤* *0.01). As evidenced by a heat map projection of the gene expression profiles (Figures 5c and S4), this filter identified 166 defence‐related genes with primed *Hpa* responsiveness in *nrpe1* and/or repressed *Hpa* responsiveness in *ros1* (Data S1). Of these 166 genes, 46 were altered in *Hpa* responsiveness only, whereas 120 showed a combination of differential expression between mock‐treated plants and differential responsiveness to *Hpa* (Figure 5b). Interestingly, in comparison to all other gene sets, the genes displaying differential *Hpa* responsiveness showed the highest proportion of gene ontology (GO) terms ‘Systemic Acquired Resistance’ and ‘Salicylic Acid Biosynthetic Process’ (Figure 5b). This outcome supports our notion that the resistance phenotypes of *nrpe1* and *ros1* are predominantly based on changes in defence gene responsiveness, rather than changes in constitutive gene expression.

### The majority of ROS1‐ and/or NRPE1‐controlled defence genes is not associated with ROS1‐ and/or NRPE1‐dependent DNA methylation in their promoter regions

**Figure 6:**
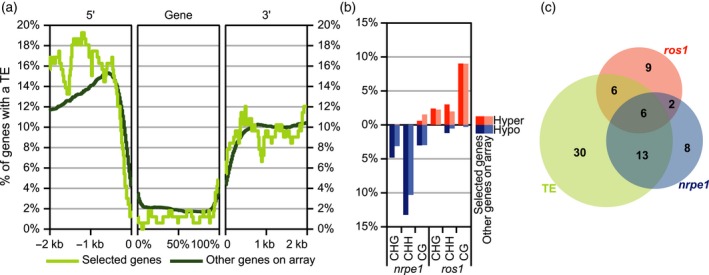
Transposable element (TE) occurrence and DNA methylation features in 166 defence genes whose responsiveness is primed in *nrpe1* and/or repressed in *ros1*. (a) Relative TE occurrence in the selection of 166 genes compared to other genes considered in the transcriptome analysis (genes on array). For the 2 kb upstream regions (5′; relative to transcriptional start site) and the 2 kb downstream regions (3′; relative to poly‐adenylation site), 100 windows of 20 bp were used; for gene body regions, 100 windows of 1% of the gene length were used. (b) Relative occurrence of differentially methylated cytosines (DmCs) in 2 kb gene promoter regions of *nrpe1* and *ros1*. Dark shades: DmC frequencies within the selection of 166 *Hpa*‐responsive genes with augmented induction in *nrpe1* and/or repressed induction in *ros1* during *Hpa* infection. Light shades: DmC frequencies in all other genes considered in the transcriptome analysis. Shown are promoters with at least three differentially methylated DmCs in *nrpe1* or *ros1*, relative to Col‐0. Results are based on publically available bisulfite sequencing data of *nrpe1* and *ros1* (Qian *et al*., 2012; Stroud *et al*., 2013). (c) Venn diagram representing a selection of the 166 gene promoters (2 kb) that contain one or more TEs (green), have at least three hyper‐methylated cytosines in the *ros1* mutant (blue), and have at least three hypo‐methylated cytosines in the *nrpe1* mutant (red).

In subsequent analyses, we focused on the selection of 166 defence‐related genes that are primed by DNA hypo‐methylation and/or repressed by DNA hyper‐methylation. First, we determined reproducibility of these microarray results by profiling transcript accumulation of four randomly selected genes in an independent experiment, using RT‐qPCR. As is shown in Figure S5, all four genes showed reproducible expression profiles to the microarray experiment. Next, we examined whether the selection of 166 defence‐related genes are regulated directly (*in cis*) or indirectly (*in trans*) by NRPE1 and ROS1‐dependent DNA (de‐)methylation. Because NRPE1 and ROS1 are known to control DNA methylation at or around transposable elements (TEs; Law and Jacobsen, 2010), we investigated whether the selection of 166 genes are enriched with nearby TEs. Using the TAIR10 annotation for known TEs, the 166 genes showed a weak enrichment of TEs within 2 kb upstream of their transcriptional start, relative to a background of all other Arabidopsis genes on the microarray (Figure 6a). By contrast, no TE enrichment was found for genic or 2 kb‐downstream regions of the 166 genes (Figure 6a). We then examined whether the TE‐enriched promoter regions are subject to NRPE1‐ or ROS1‐dependent DNA (de‐)methylation. To this end, we used publically available C‐methylomes of *nrpe1* and *ros1* (Qian *et al*., 2012; Stroud *et al*., 2013) to create a combined C‐methylome of sufficient sequence coverage (≥5 reads, 8363349 positions), before determining which of these positions are hypo‐methylated in *nrpe1* and/or hyper‐methylated in *ros1*. From this list, we selected genes with at least three differentially methylated cytosines at the same context (CG, CHG or CHH) within their 2 kb promoter region. Although the promoters of 166 defence‐related genes were marginally enriched for NRPE1‐dependent CHG and/or CHH methylation (Figure 6b), this enrichment was not statistically significant in comparison to all other genes on the microarray (χ^2^ tests; *P* = 0.3150 and 0.2837, respectively). Furthermore, the 166 gene promoters were not enriched for ROS1‐dependent hypo‐methylation. Together, this indicates that the majority of 166 defence genes are indirectly (*trans*‐)regulated by NRPE1‐ and/or ROS1‐dependent DNA (de)methylation.

### Selection of 25 defence regulatory genes that are *cis*‐regulated by NRPE1‐ and/or ROS1‐dependent DNA (de‐)methylation

To search for defence regulatory genes that are *cis*‐regulated by NRPE1‐/ROS1‐dependent DNA (de)methylation, we analysed the 2 kb gene promoter regions from the 166 NRPE1‐/ROS1‐controlled defence genes for: (i) TE presence; and (ii) occurrence of more than three hypo‐methylated cytosines in *nrpe1* and/or hyper‐methylated cytosines in *ros1*. A total of 25 gene promoters met these criteria (Figure 6c). To illustrate the DNA (de)methylation activities in these promoters, Figure S6 plots the positions of TEs and differentially methylated cytosines in *nrpe1* and *ros1*. Furthermore, using data from a recent ChIP‐sequencing study with a polyclonal antibody against native NRPE1 protein (Zhong *et al*., 2015), we show that physical binding of NRPE1 largely coincides with hypo‐methylated regions in the *nrpe1* mutant, thereby confirming localised activity by the Pol V complex. The group of 25 *cis*‐regulated genes includes genes with annotated defence regulatory activity, such as genes encoding for pattern recognition receptors (PRRs), leucine‐rich repeat (LRR) resistance proteins, CYP81D1 and DOWNY MILDEW RESISTANT 6 (Table S1), each of which has the potential to control a larger set of defence genes.

## Discussion

### Role of DNA (de)methylation processes in basal resistance

Our study has shown that DNA methylation and demethylation activities antagonistically regulate basal resistance of Arabidopsis. While previous studies reported similar effects by mutations in DNA methylation (López *et al*., 2011; Dowen *et al*., 2012; Luna *et al*., 2012; Yu *et al*., 2013; Le *et al*., 2014), we provide a comprehensive comparison of the effects of hypo‐ and hyper‐methylated DNA on basal resistance against both biotrophic (*H. arabidopsidis*) and necrotrophic pathogens (*P. cucumerina* and *A. brassicicola*). Furthermore, we show that the enhanced resistance in the hypo‐methylated *nrpe1* mutant and the enhanced susceptibility in the hyper‐methylated *ros1* mutant were linked to opposite changes in the effectiveness of callose deposition and the speed and intensity of SA‐dependent *PR1* gene induction. Hence, DNA (de)methylation determines the effectiveness of multiple layers of basal defence against biotrophic pathogens. Conversely, the enhanced susceptibility of *nrpe1* to necrotrophic *P. cucumerina* was associated with reduced responsiveness of JA‐induced *PDF1.2* and *VSP2* expression, confirming the earlier notion that NRPE1‐dependent RdDM suppresses JA‐dependent resistance via the antagonistic action of SA on JA responses (López *et al*., 2011). Surprisingly, *ros1* also displayed reduced responsiveness of JA‐induced *PDF1.2* and *VSP2* expression, despite the fact that this mutant was more resistant to both *P. cucumerina* and *A. brassicicola*. This suggests that DNA hyper‐methylation in *ros1* boosts basal resistance against necrotrophic pathogens independently of JA‐dependent defences. The unexpected finding that *nrpe1* and *ros1* are both affected in JA responsiveness might be explained by the recent discovery that RdDM regulates *ROS1* expression positively through DNA methylation of a target sequence between the TE‐containing promoter and 5′UTR of *ROS1* (Lei *et al*., 2015; Williams *et al*., 2015). As a consequence, *ROS1* is scarcely expressed in RdDM mutant backgrounds (Li *et al*., 2012), explaining why mutations in both RdDM and *ROS1* can cause similar phenotypes. For instance (Le *et al*., 2014) recently discovered that both *nrpe1* and the *rdd* (*ros1 dml2 dml3*) triple demethylase mutant have enhanced susceptible to *Fusarium oxysporum* due to lack of RdDM‐induced DNA demethylation at corresponding defence genes. By contrast, our experiments show that *nrpe1* and *ros1* display opposite resistance phenotypes to *H. arabidopsidis* and *P. cucumerina* (Figures 1, 3a and S3). Hence, basal resistance against *H. arabidopsidis* and *P. cucumerina* is not controlled by RdDM‐induced ROS1 activity, but rather by antagonistic activities of RdDM‐ and ROS1‐dependent DNA demethylation on corresponding defence genes.

### Role of DNA methylation in acquired resistance

Transgenerational acquired resistance in progeny from *Pst* DC3000‐infected Arabidopsis manifests itself as priming of SA‐dependent defences, which can be mimicked by mutations in the DNA methylation machinery (Luna *et al*., 2012). Our current study has expanded these initial observations by exploring the function of DNA (de)methylation in both SAR and TAR. The *nrpe1* mutant showed weakened within‐generation SAR against *Hpa*. However, since *nrpe1* expresses enhanced basal resistance to *Hpa* (Figure 1a), we propose that this mutant's SAR response was partially masked by its elevated level of basal resistance. The *ros1* mutant, on the other hand, was fully capable of expressing SAR (Figure 4a). Hence, DNA (de)methylation does not play a major role in within‐generation SAR. By contrast, P1 progenies from *Pst* DC3000‐infected mutant plants failed to show increased *Hpa* resistance in comparison to corresponding C1 progenies, indicating that TAR requires regulation by intact *NRPE1* and *ROS1* genes. The resistance in C1 progeny from *nrpe1* was statistically similar to that of P1 progeny from wild‐type plants (Figure 4b), thereby confirming our previous conclusion that hypo‐methylation mimics TAR (Luna and Ton, 2012; Luna *et al*., 2012). Conversely, levels of susceptibility in P1 and C1 progenies of the *ros1* mutant were significantly higher than that of C1 progeny from the wild‐type. Since *ros1* is not impaired in within‐generation SAR, we propose that Arabidopsis employs ROS1‐dependent demethylation for the imprinting of TAR in the parental generation.

The exact mechanisms by which acquired immunity is transmitted from infected parental plants to P1 progeny remains unknown. Yu *et al*. (2013) showed that *Pst* DC3000 infection of Arabidopsis represses RdDM genes, such as *AGO4*,* AGO6*,* NRPD2*, and *RDR1*, which offers a plausible explanation as to why *Pst* DC3000 induces DNA hypo‐methylation in Arabidopsis (Pavet *et al*., 2006; Dowen *et al*., 2012). It is tempting to speculate that *Pst* DC3000‐induced repression of RdDM acts in concert with ROS1, in order to mediate heritable hypo‐methylation of DNA. Comprehensive bisulfite‐sequence analysis of both vegetative tissues and reproductive tissues from healthy and *Pst* DC3000‐infected plants, as well as their resulting progenies, will be necessary to resolve the exact role of DNA (de)methylation during the imprinting, meiotic transmission and expression of TAR.

### Global regulation of defence gene expression by DNA (de)methylation

The combination of post‐translational histone modifications, histone variants and DNA methylation determines the level of compaction of chromatin (Richards, 2006; Saze, *et al*. 2012). This epigenetic regulation is especially important in genomic regions that are enriched with repetitive sequences and TEs to ensure genome stability. The chromatin state can also influence basal and pathogen‐inducible expression of defence genes by determining accessibility of the transcriptional machinery, such as transcription factors and DNA‐dependent RNA polymerase II (Pol II). To establish global impacts of DNA (de)methylation on defence gene expression, we performed whole‐genome transcriptome analysis of the DNA (de)methylation mutants at different time‐points after *Hpa* inoculation. Comparison between differentially expressed genes in *Hpa*‐inoculated wild‐type plants against all differentially expressed genes in *nrpe1* and/or *ros1* revealed that nearly half of all *Hpa*‐responsive genes (49%) are under direct or indirect control by DNA (de)methylation processes (Figure 5a). This outcome shows that the pathogenesis‐related transcriptome of Arabidopsis is under substantial and global regulation by DNA (de)methylation. Next, we focused on the patterns of gene expression that could explain the resistance phenotypes of *nrpe1* and *ros1* to *Hpa*. We reported that the 166 genes with increased *Hpa* responsiveness in the more resistant *nrpe1* mutant and/or decreased *Hpa* responsiveness in the more susceptible *ros1* mutant were more strongly enriched with GO terms ‘Systemic Acquired Resistance’ and ‘Salicylic Acid Biosynthetic Process’ than the 136 *Hpa*‐inducible genes, whose expression was only altered in mock‐treated *nrpe1* and *ros1* (Figure 5b). This indicates that the resistance phenotypes of *nrpe1* and *ros1* are predominantly caused by changes in responsiveness of defence genes. We therefore conclude that DNA (de)methylation regulates transcriptional responsiveness of SA‐dependent defence genes on a genome‐wide scale.

**Figure 7:**
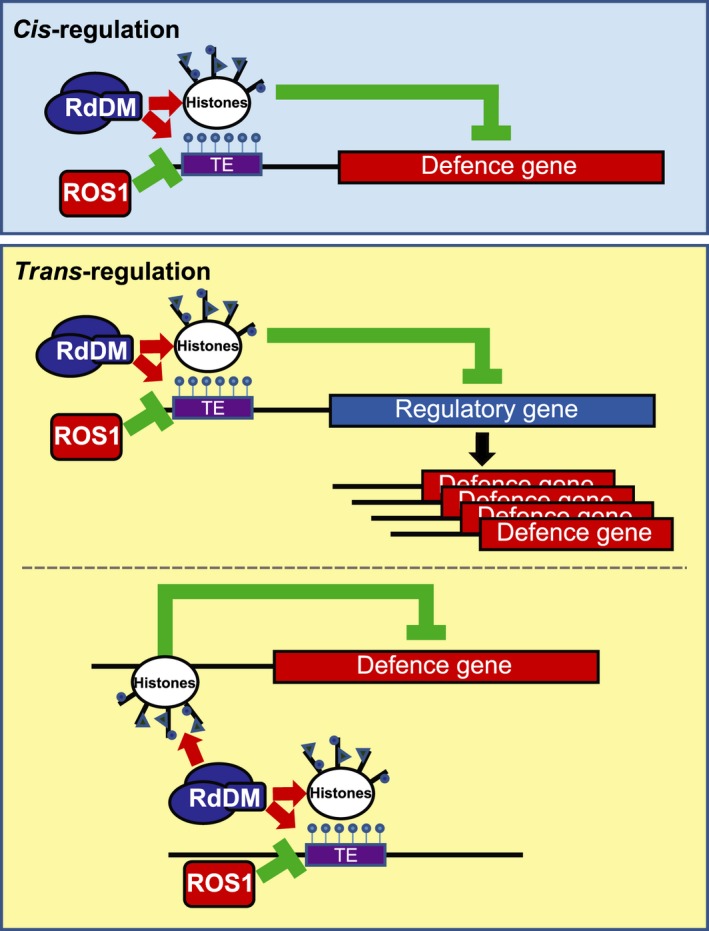
Model of *cis*‐ and *trans*‐regulation of defence gene responsiveness by DNA (de)methylation. Responsiveness of defence genes can be *cis*‐regulated via RNA‐directed DNA methylation (RdDM; blue) and/or ROS1‐mediated DNA demethylation (red) of nearby DNA regions, such as transposable elements (TEs; purple). *Trans*‐regulation of defence genes that are not associated with nearby DNA methylation can be achieved via different mechanisms. Apart from indirect regulation by *cis*‐controlled regulatory genes (top), chromatin remodellers in the RdDM protein complex can cross‐link with distant genomic regions and influence post‐translational histone modifications at distal genes that are not associated with DNA methylation. Red arrows indicate stimulation of DNA methylation and/or post‐translational histone modifications (blue triangles and circles) by the RdDM complex. Green lines indicate repression of DNA methylation by ROS1, or transcriptional repression by post‐translational histone modifications. The black arrow indicates stimulation of defence gene induction by defence regulatory proteins.

DNA (de)methylation could regulate defence gene responsiveness via *cis*‐ and *trans*‐regulatory mechanisms (Figure 7). To explore a possible *cis*‐regulatory role of NRPE1/ROS1‐dependent DNA (de)methylation, we examined TE occurrence and NRPE1‐binding sequences in the selection of 166 defence‐related gene promoters that are antagonistically controlled by *NRPE1* and *ROS1*. Surprisingly, we only detected relatively weak over‐representation of TEs in the 166 gene promoters compared to the genomic background average (Figure 6a), even though RdDM and ROS1 are both known to act on TE‐containing intergenic sequences (Chan *et al*., 2005). Moreover, the 166 gene promoters were not statistically enriched with sequences that are de‐methylated in *nrpe1* and/or hyper‐methylated in *ros1* (Figure 6b). We therefore conclude that the influence of NRPE1/ROS1‐dependent (de)methylation on defence gene responsiveness is predominantly enacted by *trans*‐regulatory mechanisms.

There are different mechanisms by which DNA (de)methylation can regulate defence gene induction in *trans* (Figure 7). For instance a small number of signalling genes that are directly *cis*‐regulated by DNA (de)methylation can control induction of a much larger group of defence genes. In fact, of the 166 genes with altered *Hpa* responsiveness, we identified only 25 genes whose promoters contain a TE and show evidence for NRPE1‐/ROS1‐dependent DNA (de)methylation and/or binding to the NRPE1 unit of Pol V (Figures 6c and S6). Since their responsiveness to *Hpa* is influenced by mutations in *NRPE1* and *ROS1* (Figure 5c), it is plausible that these 25 genes are *cis*‐regulated by NRPE1‐/ROS1‐dependent DNA (de)methylation. This group includes genes with annotated regulatory activity in plant defence (Figure S6 and Table S1), such as PRR and R proteins, which can initiate downstream defence pathways and activate a wider range of defence genes. An alternative mechanism by which DNA (de)methylation can *trans*‐regulate defence genes is through influencing chromatin density at distant genome loci. Like DNA methylation, chromatin density has been reported to have long‐lasting impacts on gene expression and responsiveness (Vaillant and Paszkowski, 2007). Furthermore, both mechanisms are highly co‐regulated, since Arabidopsis mutants affected in DNA methylation are also altered in post‐translational modifications of histones that mark chromatin density (Law and Jacobsen, 2010). Previous studies have shown that priming of defence genes is associated with post‐translational modifications of histone proteins in their promoter regions, such as triple‐methylation of lysine 4 and acetylation of lysine 9 in the tail of histone H3 (Jaskiewicz *et al*., 2011; López *et al*., 2011; Luna *et al*., 2012). Hence, chromatin structure can act as a *cis*‐regulatory mechanism of defence gene priming. Interestingly, however, some defence gene promoters are subject to histone modifications in primed plants, even though these regions are not methylated at the DNA level (López *et al*., 2011; Slaughter *et al*., 2012). Under these premises, it is tempting to speculate that the Pol V‐associated chromatin‐remodelling complex (Zhong *et al*., 2012; Zhu *et al*., 2013; Liu *et al*., 2014) can increase chromatin density at multiple chromosomal positions via cross‐linking distant loci (Figure 7). In this scenario, it is possible that Pol V‐dependent DNA methylation at specific TEs influences chromatin structure at genomically distant defence genes. This mechanism would enable *trans*‐regulation of defence genes by RdDM, and explain earlier reports that TAR is associated with histone modifications at defence genes that are not associated with nearby DNA methylation (Luna *et al*., 2012; Slaughter *et al*., 2012). Chromatin immuno‐precipitation of NRPE1 followed by chromosome conformation capture analysis (‘ChIP‐loop’) and next‐generation sequencing is one future approach which could resolve whether the Pol V complex indeed cross‐links *cis*‐methylated DNA regions with *trans*‐regulated defence genes during pathogen attack.

## Experimental procedures

### Plant material

Seeds of *ros1‐4* (SALK_135293), *ros3* (SALK_022363C) and *cmt3‐11* (SALK_148381) were obtained from the Col‐0 Salk T‐DNA collection (Alonso *et al*., 2003) and verified to be homozygous for the T‐DNA insertion (Figure S1a); *nrpe1‐11* (SALK_029919) and *drd1‐6* (Kanno *et al*., 2004) were kindly provided by P. Vera and D.C. Baulcombe respectively. Knock‐down of ROS1 and NRPE1 gene expression was confirmed by RT‐qPCR (Figure S1b). Seeds of the F4 of *ddm1‐2* (Vongs *et al*., 1993) were kindly provided by V. Colot. Growth conditions are detailed in Methods S1.

### Basal resistance assays

To quantify basal resistance against *H. arabidopsidis* (isolate WACO9), seedlings were grown for 3 weeks before spray inoculation with a suspension containing 10^5^ conidiospores ml^−1^, as described in Methods S1. For basal resistance assays to *P. cucumerina* and *A. brassicicola*, fungi was grown in darkness at room temperature on full‐strength PDA plates and half‐strength PDA agar plates containing 20 g L^−1^ sucrose and 30 g L^−1^ CaCO_3_, respectively. Fungal spores were collected by scraping water‐flooded plates. Plants (4.5‐week‐old) were inoculated by applying 6 μl‐droplets (10^6^ spores ml^−1^) onto four leaves of similar physiological age per plant. Inoculated plants were kept at 100% humidity until scoring disease or sample collection (as described in Methods S1). To investigate defence responsiveness to JA, 4.5‐week‐old Arabidopsis plants were sprayed with 0.016% v/v ethanol and 0.01% v/v Silwet L‐77 (Vac‐In‐Stuff; LEHLE Seeds, Round Rock, TX, USA; catalogue number VIS‐30) in dH_2_O with (treatment) or without (mock) 0.1 mm (±)‐JA (Sigma, St. Louis, MO, USA catalogue number J2500).

### SAR assays

Systemic acquired resistance was induced in 4.5‐week‐old plants, using avirulent *Pseudomonas syringae* pv. *tomato* DC3000, carrying *avrRpm1*. Four lower leaves per plant were pressure infiltrated using with 10 mm MgSO_4_ with or without (mock) 10^7^ cfu ml^−1^ *Pst* DC3000 (*avrRpm1*), using a needleless syringe. Plants were challenged 3 days later by spray inoculation with *H. arabidopsidis* (10^5^ conidiospores ml^−1^). At 5 dpi, distal leaves from infiltrated leaves were collected for trypan blue staining. For TAR assays, plants were grown under long day conditions (16 h light/8 h dark, 21°C, 80% relative humidity, light intensity 100–140 μmol sec^−1^ m^−2^) and spray‐inoculated at 21, 28 and 35 days after germination with 10 mm MgSO_4_ containing 10^8^ cfu ml^−1^ *Pst* DC3000 (P0; diseased) or 10 mm MgSO_4_ (C0; mock). Progeny from P0 and C0 plants (P1 and C1) were grown for 3 weeks and challenged by spray‐inoculating *H. arabidopsidis* (10^5^ conidiospores ml^−1^). At 6 dpi, leaves were collected for trypan blue staining. All staining procedures are detailed in the Methods S1. Bacteria were grown overnight at 28°C in liquid KB or LB medium containing 50 mg L^−1^ rifampicin and, for *Pst* DC3000(*avrRpm1*), 50 mg L^−1^ kanamycin.

### RNA extraction and RT‐PCR

Samples were snap‐frozen in liquid nitrogen and ground to a fine powder. RNA was extracted using modified guanidinium thiocyanate–phenol–chloroform extraction methods, as detailed in the Methods S1. To remove residual DNA, samples were treated with DNase I (Promega, Fitchburg, WI, USA) for 30 min at 37°C. First strand cDNA synthesis and RT‐PCR analysis were performed as described in the Methods S1.

### Microarray analysis

Col‐0, *nrpe1* and *ros1* plants were grown as described for *Hpa* basal resistance assays. Samples were taken at 48 and 72 hpi by pooling leaves from 10 to 12 seedlings per treatment from the same pot. Four biologically replicated samples were used to represent each treatment/genotype combination. RNA was extracted, as described above, and analysed using Affymetrix Arabidopsis Gene 1.0 ST arrays, according to manufacturer's instructions. Details of array processing and statistical analysis using R‐packages oligo (Carvalho and Irizarry, 2010) and Limma (Smyth, 2004; Ritchie *et al*., 2015) are included in Methods S1. Data have been deposited at EMBL (E‐MTAB‐3963). GO‐term over‐representation analysis was performed using GOrilla (Eden *et al*., 2009).

### Analysis of sequencing data

Bisulfite sequencing reads from two previous studies (Qian *et al*., 2012; Stroud *et al*., 2013) were downloaded from NCBI's SRA (accession numbers SRR353936‐SRR353939, SRR534177, SRR534182 and SRR534193). Processing of raw sequence data is detailed in Methods S1. ChIP‐seq data from (Zhong *et al*., 2015) were downloaded from NCBI's GEO (series number GSE61192).

## Supporting information


**Figure S1.** Genetic characterization of selected mutants.

**Figure S2.** Repeats of pathogenicity assays to determine basal resistance in DNA (de)methylation mutants against *H. arabidopsidis*.

**Figure S3.** Basal resistance phenotypes of Col‐0, *nrpe1* and *ros1* to the necrotrophic fungi *Plectosphaerella cucumerina* and *Alternaria brassicicola*.

**Figure S4.** Transcript levels of 166 *Hpa‐*inducible genes with augmented induction in *nrpe1* and/or repressed induction in *ros1*.

**Figure S5.** Microarray validation of transcriptional profiles from an independent *Hpa* experiment.

**Figure S6.** Schematic overview of the 2 Kb promoter regions of 25 defence‐related genes that are *cis*‐regulated by DNA (de)methylation.

**Table S1.** Annotations of 25 candidate defence regulatory genes that are *cis*‐regulated by NRPE1‐ and/or ROS1‐dependent DNA (de)methylation.

**Methods S1.** Details about plant growth conditions, basal resistance assays, staining procedures and resistance classifications, nucleic acid extractions and qPCR, primer sequences, microarray analysis, and analysis of sequencing data.

**Data S1.** Gene transcripts showing statistically significant differences in normalized hybridization signal (Affymetrix Arabidopsis Gene 1.0 ST arrays) between Col‐0, *nrpe1*, and *ros1* at 48 and 72 h after mock or *Hpa* inoculation.
