# Supplementary material for: The role of DNA (de)methylation in immune responsiveness of Arabidopsis
Source: Plant J. 2016 Sep 7;88(3):361–74. doi: 10.1111/tpj.13252 (PMC5132069; doi:10.1111/tpj.13252)
Supplement: Supplementary file 1 — Figure S1. Genetic characterization of selected mutants. [file TPJ-88-361-s001.pdf]

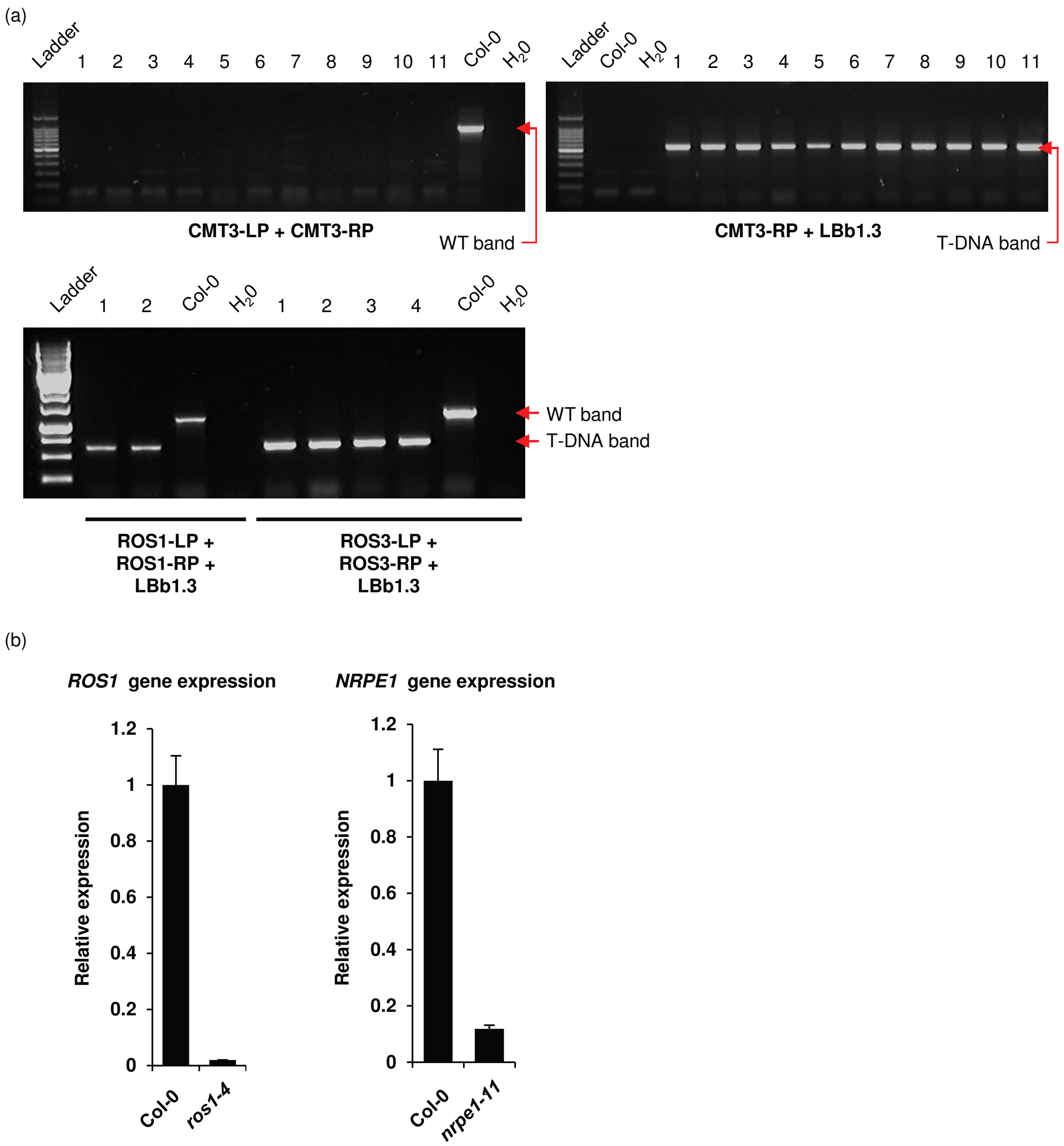

Figure S1: Genetic characterization of selected mutants. (a) Genotyping of *cmt3*, *ros1* and *ros3* mutants. (b) Expression levels of *ROS1* (left) and *NRPE1* (right) in the *ros1* and *nrpe1* mutant, respectively, relative to wild-type Col-0. Primers used in (a) and (b) are described in Methods S1.
