# Supplementary material for: The role of DNA (de)methylation in immune responsiveness of Arabidopsis
Source: Plant J. 2016 Sep 7;88(3):361–74. doi: 10.1111/tpj.13252 (PMC5132069; doi:10.1111/tpj.13252)
Supplement: Supplementary file 2 — Figure S2. Repeats of pathogenicity assays to determine basal resistance in DNA (de)methylation mutants against H. arabidopsidis. [file TPJ-88-361-s002.pdf]

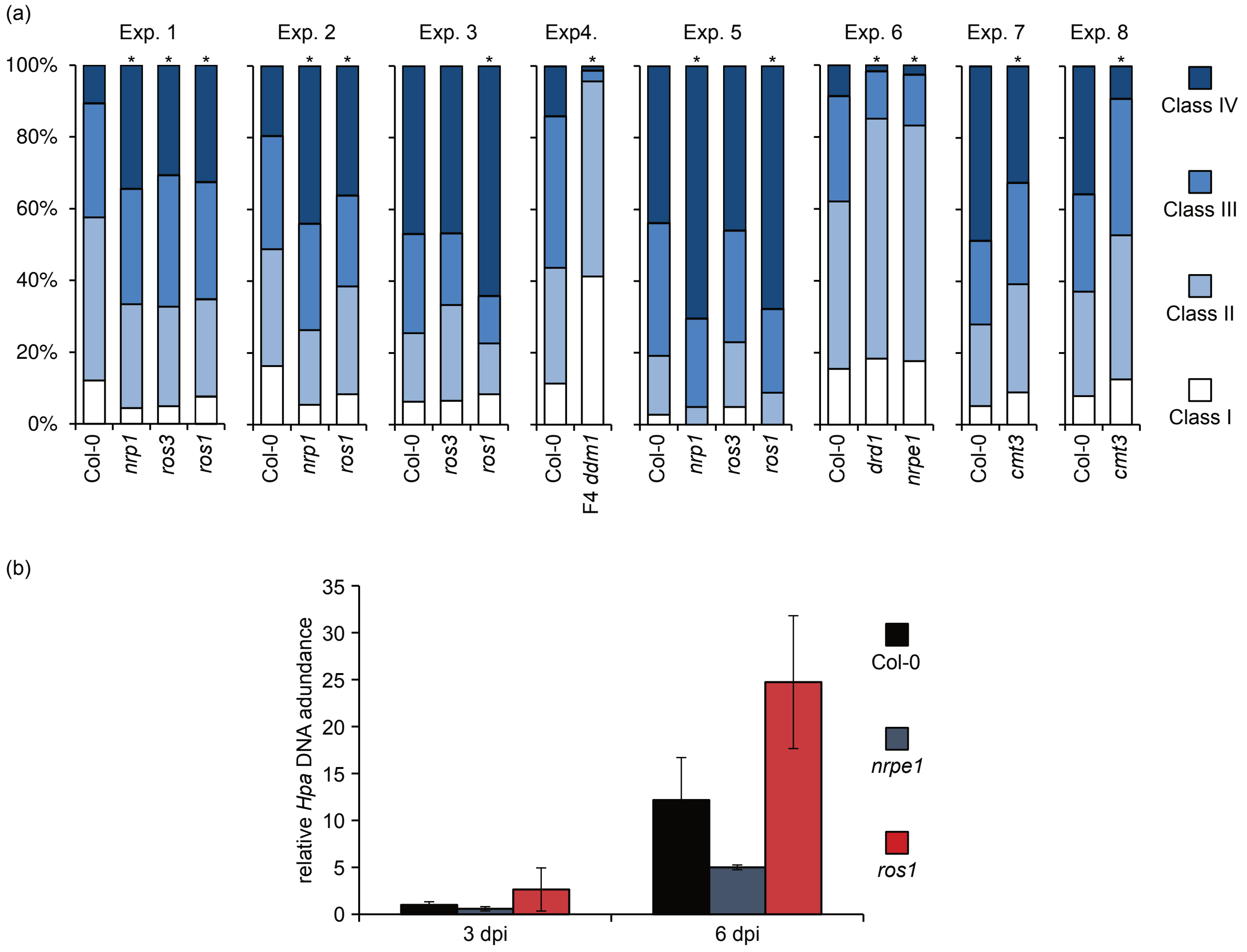

Figure S2: Repeats of pathogenicity assays to determine basal resistance in DNA (de)methylation mutants against *Hyaloperonospora arabidopsidis* (*Hpa*). (a) Trypan blue visual scoring of *Hpa* infections, as in Figure 1a. (b) RT-qPCR-based quantification of the presence of *Hpa* DNA relative to Arabidopsis DNA. Infected material harvested three days post-inoculation of Col-0 was taken as reference.
