# Supplementary material for: The role of DNA (de)methylation in immune responsiveness of Arabidopsis
Source: Plant J. 2016 Sep 7;88(3):361–74. doi: 10.1111/tpj.13252 (PMC5132069; doi:10.1111/tpj.13252)
Supplement: Supplementary file 3 — Figure S3. Basal resistance phenotypes of Col‐0, nrpe1 and ros1 to the necrotrophic fungi Plectosphaerella cucumerina and Alternaria brassicicola. [file TPJ-88-361-s003.pdf]

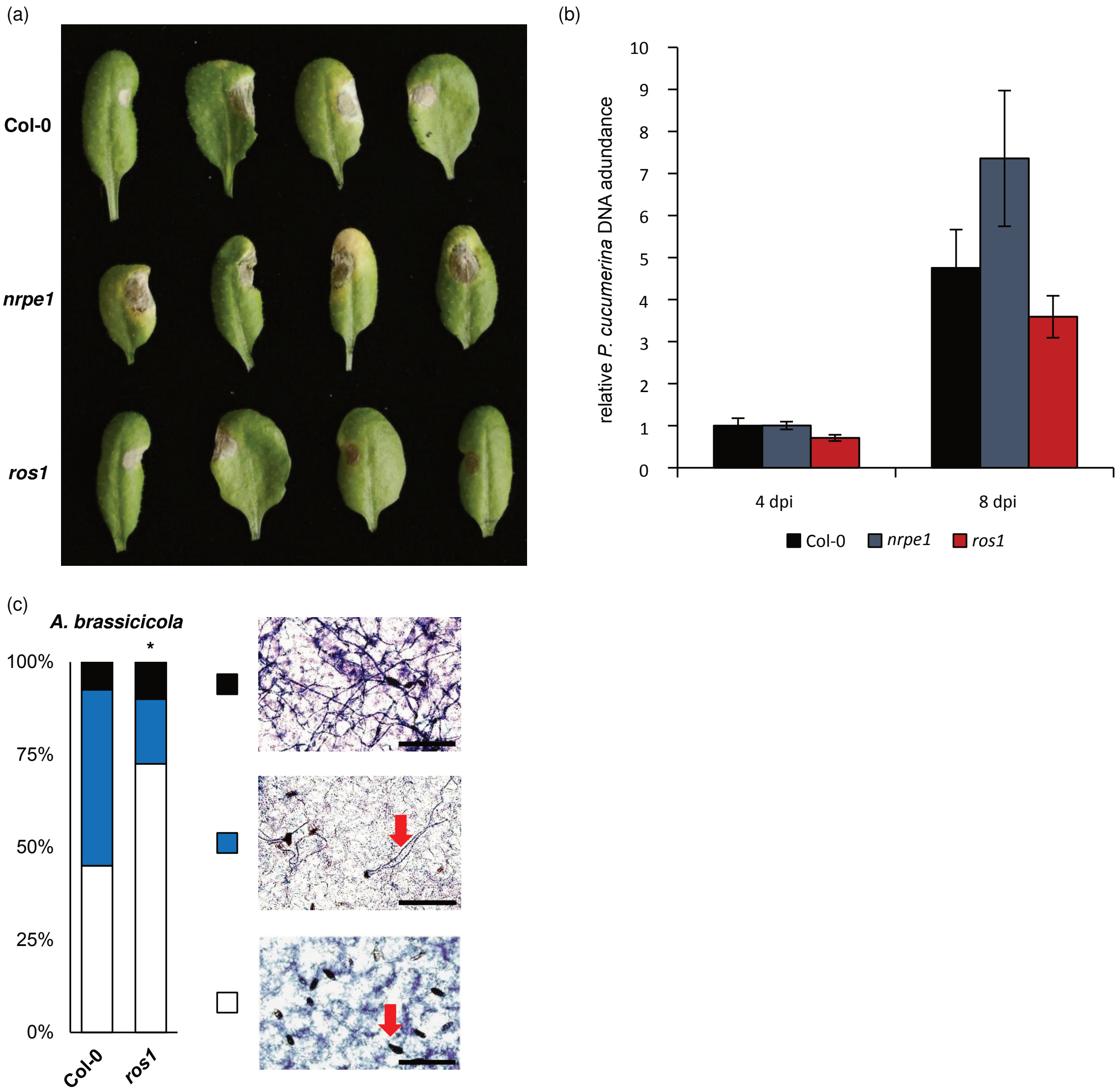

Figure S3: Basal resistance phenotypes of Col-0, *nrpe1* and *ros1* to the necrotrophic fungi *Plectosphaerella cucumerina* and *Alternaria brassicicola*. (a) Representative images of *P. cucumerina* lesion formation on Col-0, *nrpe1* and *ros1*. Quantification of diameters is presented in Figure 3a. (b) RT-qPCR-based estimation of the presence of *P. cucumerina* DNA relative to Arabidopsis DNA. Values are presented with Col-0 four days post-inoculation (dpi) as reference. (c) Susceptibility of *ros1* and Col-0 to *Alternaria brassicicola*. Plants were drop-inoculated at five weeks post germination with 6 µl droplets of a 10^6 spores ml^{-1} spore suspension. At 14 days post inoculation, disease symptoms were assessed in a total of 40 leaves from 10 plants after trypan blue staining. Classes are defined as follows; lowest: no hyphal colonisation of the leaf – arrow indicates spore; intermediate: low/intermediate density of hyphal colonisation – arrow indicates hypha; highest: dense hyphal colonisation with a presence of necrotic lesions. The asterisk indicates a statistically significant difference in distribution of classes (χ^2 test, $p < 0.05$). Scale bars: 100 µm. *A. brassicicola* spores were prepared as described for *P. cucumerina* except *A. brassicicola* was grown on PDA-$CaCO_3$ plates.
