# Supplementary material for: The role of DNA (de)methylation in immune responsiveness of Arabidopsis
Source: Plant J. 2016 Sep 7;88(3):361–74. doi: 10.1111/tpj.13252 (PMC5132069; doi:10.1111/tpj.13252)
Supplement: Supplementary file 4 — Figure S4. Transcript levels of 166 Hpa‐inducible genes with augmented induction in nrpe1 and/or repressed induction in ros1. [file TPJ-88-361-s004.pdf]

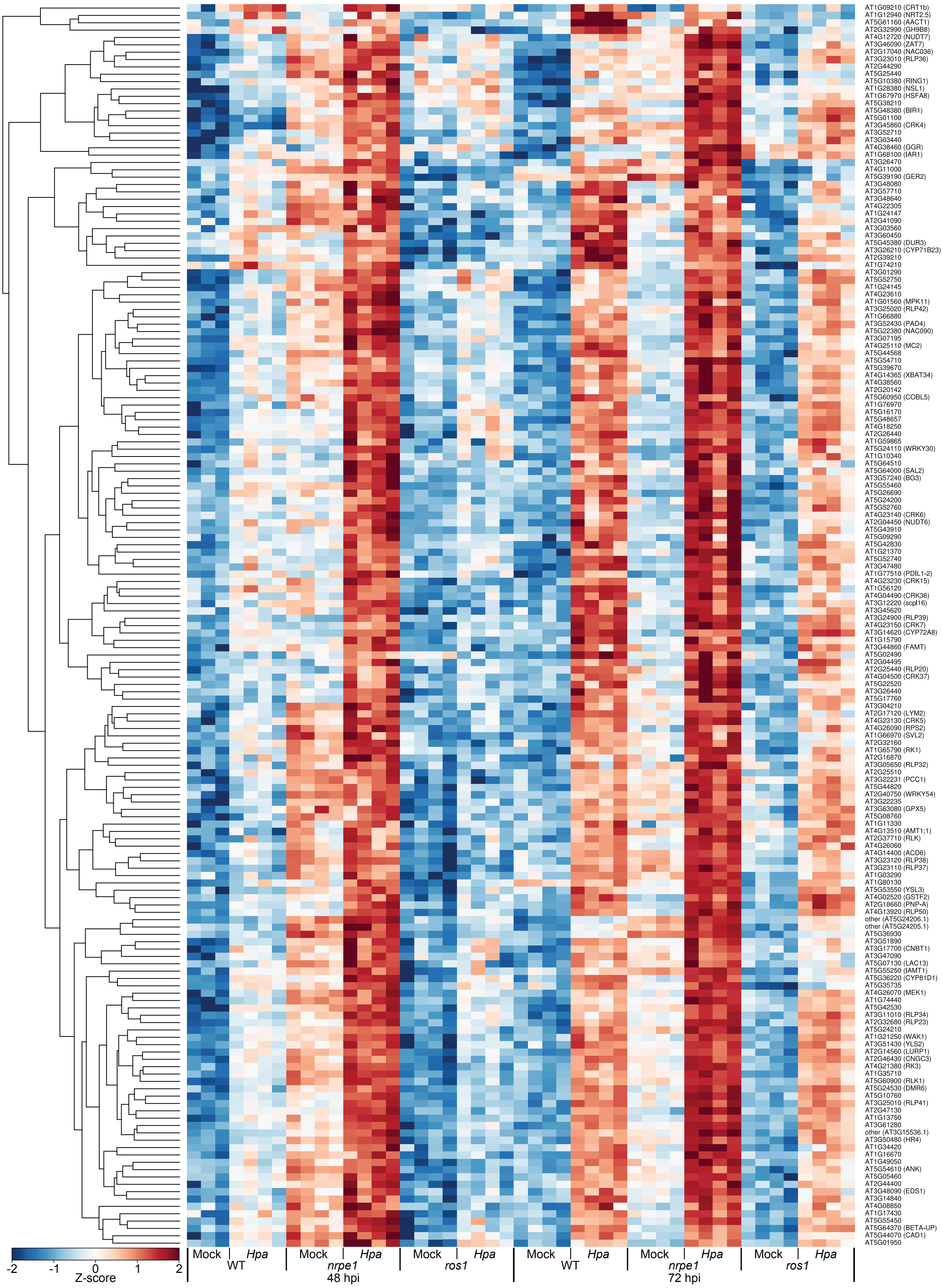

Figure S4: Transcript levels of 166 *Hpa*-inducible genes with augmented induction in *nrpe1* and/or repressed induction in *ros1*. Genes were selected when differentially expressed between *ros1* and *nrpe1*, as well as between Col-0 and *ros1*, and/or between Col-0 and *nrpe1*, at either 48 or 72 hours post-inoculation (hpi). Heat map projections represent Z-scorse of transcript levels.
