# Supplementary material for: The role of DNA (de)methylation in immune responsiveness of Arabidopsis
Source: Plant J. 2016 Sep 7;88(3):361–74. doi: 10.1111/tpj.13252 (PMC5132069; doi:10.1111/tpj.13252)
Supplement: Supplementary file 5 — Figure S5. Microarray validation of transcriptional profiles from an independent Hpa experiment. [file TPJ-88-361-s005.pdf]

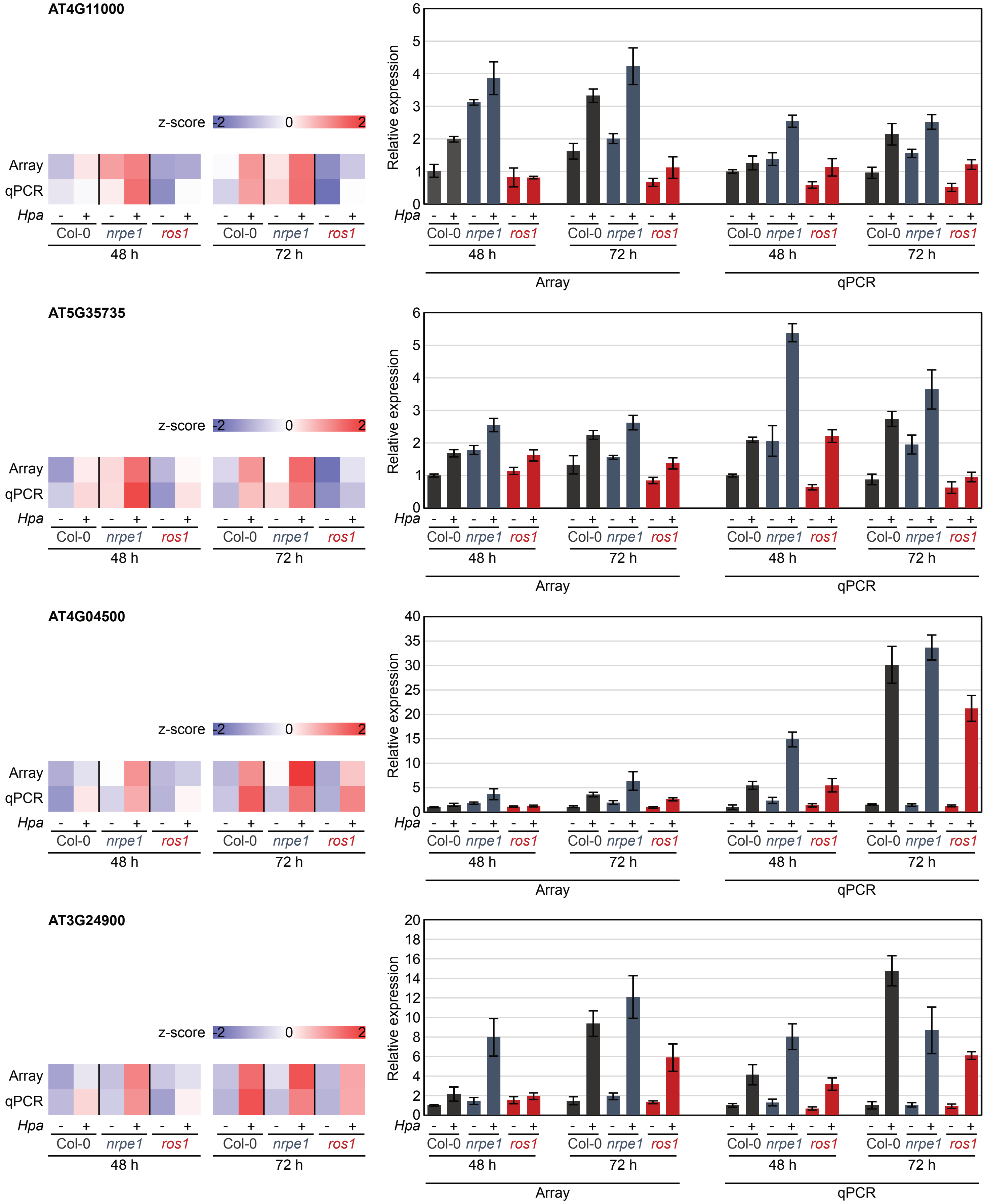

Figure S5: Micro-array validation of transcriptional profiles from an independent *Hpa* experiment. Heat maps visualise the z-scores derived from the RMA-normalised expression values from the array and the -ΔCt values from RT-qPCR experiments. Bar graphs show expression relative to mock-inoculated Col-0 at 48 hours post inoculation (hpi).
