# Supplementary material for: The role of DNA (de)methylation in immune responsiveness of Arabidopsis
Source: Plant J. 2016 Sep 7;88(3):361–74. doi: 10.1111/tpj.13252 (PMC5132069; doi:10.1111/tpj.13252)
Supplement: Supplementary file 6 — Figure S6. Schematic overview of the 2 Kb promoter regions of 25 defence‐related genes that are cis‐regulated by DNA (de)methylation. [file TPJ-88-361-s006.pdf]

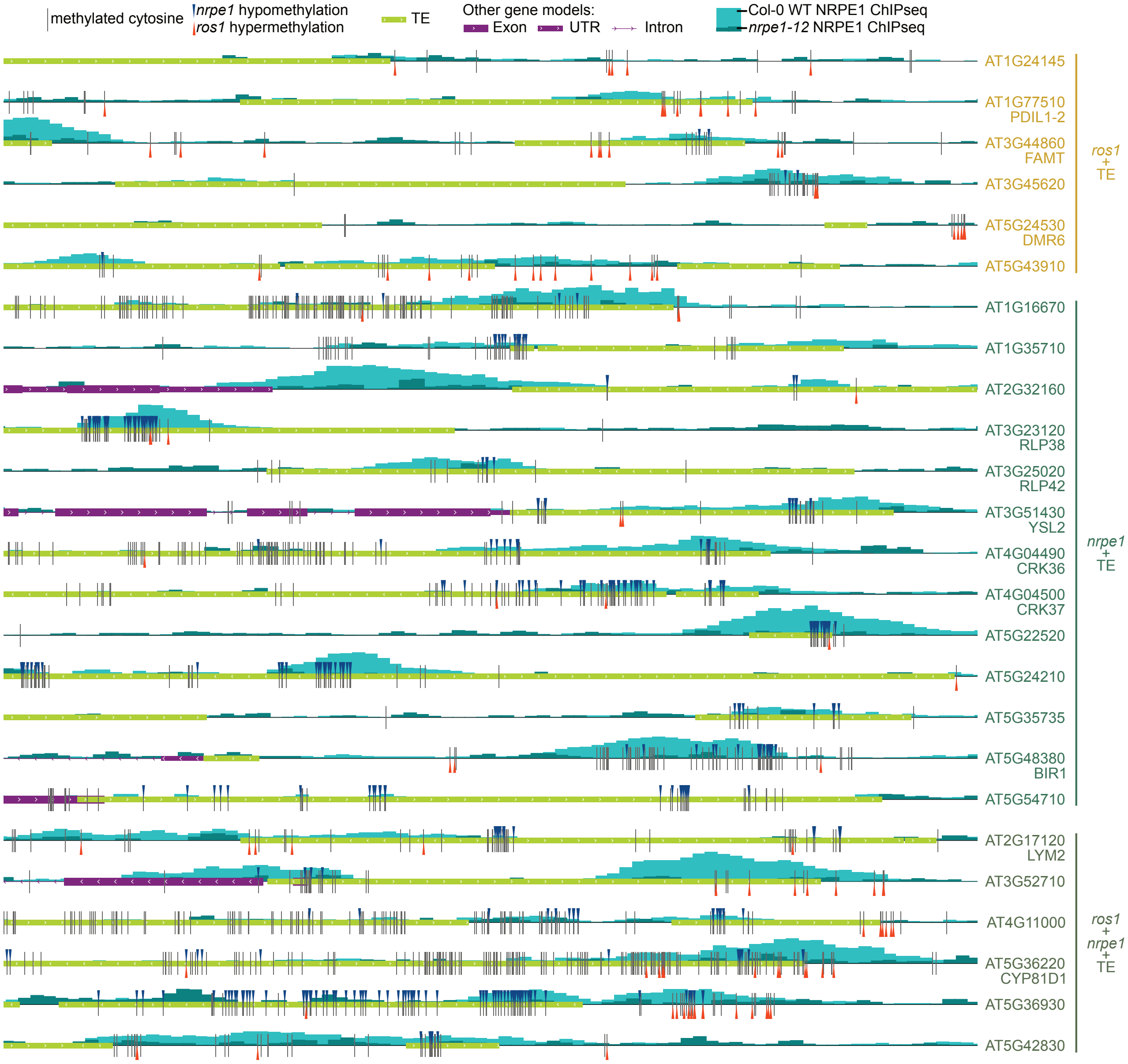

Figure S6: Schematic overview of the 2 kb defence gene promoters that contain one or more TEs, have three or more hyper-methylated cytosines in the *ros1* mutant, and/or have three or more hypo-methylated cytosines in the *nrpe1* mutant. Cytosine methylation is indicated by vertical ticks/lines. Grey vertical lines: cytosines for which ≥20% of the reads indicate methylation (i.e. C to T conversion after bisulfite treatment); Blue ticks: cytosines that are hypo-methylated in *nrpe1*; Red ticks: cytosines that are hyper-methylated in *ros1*. Green horizontal bars: TEs. Purple bars: other gene models that fall into the 2 kb region upstream from the transcriptional start site of the gene. Teal histograms indicate association with NRPE1 as deternined by ChIP-seq (Zhong et al., 2015); the superimposed darker colour indicates background levels (ChIP-seq data obtained from the *nrpe1-12* mutant).
