# Supplementary material for: The role of DNA (de)methylation in immune responsiveness of Arabidopsis
Source: Plant J. 2016 Sep 7;88(3):361–74. doi: 10.1111/tpj.13252 (PMC5132069; doi:10.1111/tpj.13252)
Supplement: Supplementary file 7 — Table S1. Annotations of 25 candidate defence regulatory genes that are cis‐regulated by NRPE1‐ and/or ROS1‐dependent DNA (de)methylation. [file TPJ-88-361-s007.docx]

**Table S1: Annotations of 25 candidate defence-regulatory genes that are *cis*-regulated by NRPE1- and/or ROS1-dependent DNA (de-)methylation.**

| Gene | Annotation |
| --- | --- |
|  |  |
| AT1G24145 | unknown protein |
| AT1G77510 | protein disulfide isomerase-like (PDIL) protein; PDI6 |
| AT3G44860 | farnesoic acid carboxyl-O-methyltransferase; FAMT |
| AT3G45620 | DWD motif protein; may be involved in formation of CUL4-based E3 ubiquitin ligase |
| AT5G24530 | 2OG-Fe(II) oxygenase; DOWNY MILDEW RESISTANT 6 (DMR6) |
| AT5G43910 | pfkB-like carbohydrate kinase family protein |
|  |  |
| AT1G16670 | Protein kinase superfamily protein |
| AT1G35710 | Leucine-rich repeat receptor-like protein kinase family protein |
| AT2G32160 | S-adenosyl-L-methionine-dependent methyltransferases superfamily protein |
| AT3G23120 | Receptor like protein 38 |
| AT3G25020 | Receptor like protein 42 |
| AT3G51430 | strictosidine synthase like protein; YELLOW-LEAF-SPECIFIC GENE 2 (YLS2) |
| AT4G04490 | Cystein-rich receptor-like kinase (RLK) 36 |
| AT4G04500 | Cystein-rich receptor-like kinase (RLK) 37 |
| AT5G22520 | unknown protein |
| AT5G24210 | alpha/beta-Hydrolases superfamily protein |
| AT5G35735 | Auxin-responsive family protein |
| AT5G48380 | receptor-like kinase; BIR1 |
| AT5G54710 | Ankyrin repeat family protein |
|  |  |
| AT2G17120 | LYSM-containing receptor protein 1 |
| AT3G52710 | unknown protein |
| AT4G11000 | Ankyrin repeat family protein |
| AT5G36220 | Cyp81D1 |
| AT5G36930 | Disease resistance protein (TIR-NBS-LRR class) family |
| AT5G42830 | HXXXD-type acyl-transferase family protein |
