# Supplementary material for: The role of DNA (de)methylation in immune responsiveness of Arabidopsis
Source: Plant J. 2016 Sep 7;88(3):361–74. doi: 10.1111/tpj.13252 (PMC5132069; doi:10.1111/tpj.13252)
Supplement: Supplementary file 8 — Methods S1. Details about plant growth conditions, basal resistance assays, staining procedures and resistance classifications, nucleic acid extractions and qPCR, primer sequences, microarray analysis, and analysis of sequencing data. [file TPJ-88-361-s008.docx]

### Supplemental Methods

### Plant growth conditions

Seeds were stratified in water at 4°C in darkness for 3-5 days before being sown on soil or on *Jiffy‑7* peat pellets (Jiffy). Plants were grown under an 8.5 hour daily photo period (100‑140 µmol s^‑1^ m^‑2^ light intensity) at 21°C and 80% relative humidity, unless indicated otherwise.

### Basal resistance assays

For assays to quantify basal resistance against *H. arabidopsidis* (*Hpa*), seedlings were grown for 3 weeks before spray-inoculation with a suspension of 10^5^ conidiospores ml^-1^ from *H. arabidopsidis* isolate WACO9. The pathogen was maintained on hyper-susceptible Ws-NahG seedlings and transferred to fresh seedlings on a weekly basis. Spores were harvested by suspending sporulating seedlings in water and filtering the suspension though Miracloth (Merck Millipore). Spray-inoculated plants were left to air-dry for 30-60 minutes and then kept at 100% humidity until sampling for trypan-blue staining or DNA extraction (see below). In the analysis of the defence associated phenotype by trypan blue staining, leaves (~200 from 35 plants) were microscopically analysed and assigned to different colonization classes; class I: no hyphal colonisation; class II: hyphal colonisation but no sporulation; class III: hyphal colonisation with conidiophores; class IV: extensive hyphal colonisation with conidiophores and oospores. For the analysis of the defence phenotype by quantification of *Hpa* biomass using q-PCR, 12 infected seedlings were collected at 3 or 6 days post inoculation (dpi) in 2 ml Eppendorf tubes with metallic beds, frozen in liquid nitrogen and stored at -80°C. DNA was extracted by CTAB and analysed by q-PCR (see below). To determine effectiveness of callose deposits, seedlings were collected at 48 hours after inoculation for aniline blue/calcofluor white double staining (see below). *Hpa*-callose interactions were categorised into four different classes of defence effectiveness; class I: spores completely blocked by callose before or shortly after germination; class II: spores not affected by callose deposition, showing extensive growth, and able to penetrate callose at multiple sites.

Basal resistance against *Plectosphaerella cucumerina* (*P. cucumerina*) was assessed by measuring the lesion diameter of the infection or by fungal biomass quantification by q-PCR. Plants (4.5 week-old) were inoculated by applying 6 µl-droplets (10^6^ spores ml^-1^) onto four leaves of similar physiological age per plant. At 8 dpi, lesion diameters were measured for each individual plant before analysing averaged mean values from 27 plants per genotype for statistical differences. For fungal biomass quantification leaf discs around the infection point were taking using a 7.5mm-diameter core bore. Eight replicates each containing 12 discs from 3 different plants (4 discs/plant and 3 plants/replicate) were collected at 4 and 8 dpi in 2ml Eppendorf tubes with metallic beads, frozen in liquid nitrogen and stored at -80°C. DNA was extracted by CTAB and analysed by q-PCR (see below).

Colonization by *A. brassicicola* was assessed at 14 dpi by collecting 40 leaves from 10 different plants for trypan blue-staining. Stained leaves were examined microscopically and assigned to 3 different classes of hyphal colonization and cell death: Class I, no hyphal growth from fungal spores; Class II, limited number of spores producing hyphal colonization; Class III, extensive hyphal colonization with large areas of cell death (necrosis). Differences in class distributions were analysed for statistical difference by Pearson’s Chi-square or Fisher exact tests, using SPSS software (v19).

### Staining procedures

For trypan blue staining, samples were collected in 100% ethanol and then transferred to a staining solution of 1 part lactophenol-trypan blue solution (0.067% w/v trypan blue (Sigma-Aldrich), 33% w/v phenol (VWR Chemicals), 33% v.v glycerol (Fisher Scientific) and 33% v.v DL-lactic acid (Fluka) in dH_2_O) and 2 parts 100% ethanol. Tubes containing samples were incubated in boiling water twice for 1 minute with a 5 minute interval at room temperature, and then left at room temperature to incubate for 3-5 hours. Samples were stored in 60% w/v chloral hydrate (Sigma-Aldrich) at least overnight before *Hpa* colonization was scored. For aniline blue/calcofluor white double staining, samples were cleared for at least 24 hours in 100% ethanol and incubated for 30 minutes in 0.07 mM phosphate buffer (pH 9) before staining. Samples were then incubated in a 4:1 mixture of 0.05% w/v aniline blue (Sigma-Aldrich) in 0.07 M phosphate buffer (pH 9) and 0.025% w/v calcofluor white M2R (Sigma-Aldrich) in 0.1 M Tris-HCl (pH 7.5) for 15 minutes, before overnight incubation in 0.5% w/v aniline blue (Sigma-Aldrich) in 0.07 mM phosphate buffer (pH 9). Stained leaves were placed onto microscope slides in 0.05% w/v aniline blue (Sigma-Aldrich) in 0.07 mM phosphate buffer (pH 9) and imaged on an Olympus BX 51 microscope using an XF02-2 filter (excitation 330 nm, emission 400 nm).

### Nucleic acid extraction and RT-q-PCR

DNA extractions for genotyping were performed as previously described (Kasajima *et al.*, 2012). For RNA and other DNA extractions, samples were snap-frozen in liquid nitrogen and ground to a fine powder by shaking samples in 2 ml tubes with 3 x 3mm diameter steel balls (Atlas Ball and Bearings Co. Ltd.) in a mill (SPEX 8000M) for 30-45 seconds. DNA extraction for pathogen biomass quantification was performed by CTAB. Frozen samples were treated with 1ml of CTAB buffer (2% CTAB, Sigma; 100mM Tris-HCl pH 8, Melford; 1.4M NaCl, Fisher Chemical; 20mM EDTA, Melford; 1% PVP- 40, Sigma; 2µ ml^-1^ 2-Mercaptoethanol, Aldrich, was added immediately before use), homogenised and incubated for 60 minutes at 65°C. Then 1 volume of chloroform (Fisher Chemical) was added and mixed by vortex before centrifuging 8 minutes at 9500***g***. The aqueous phase was transferred to a clean tube then DNA was precipitated using 1 volume of isopropanol (Fisher Chemical) and incubating at 30 minutes at room temperature. After centrifuging for 15 minutes (16,500 ***g*** at 4°C), the pellet was washed with 70% ethanol (Fisher Chemical) and centrifuged for 5 minutes (16,500 ***g*** at 4°C). The pellets were air-dried and resuspended in water. RNA was removed by precipitation with 2M of LiCl (Sigma), incubation at 4°C overnight and centrifugation for 20 minutes (16,500 ***g*** at 4 °C). The soluble DNA was precipitated with absolute ethanol for 4 hours at -20°C and centrifuging for 20 minutes (16,500 ***g*** at 4°C). DNA pellets were washed with 70% ethanol and centrifuged for 5 minutes (16,500 ***g*** at 4°C), air-dried and resuspended in water. The DNA was quantified using a nanodrop spectrophotometer (Nanodrop 8000 Spectrophotometer, Thermo Scientific) and standardised to 200 ng μl^-1^ for all the samples. For RNA extraction, powder was vortexed for 30 seconds in 1 ml Extraction buffer [1 M guanidine thiocyanate (Amresco), 1 M ammonium thiocyanate (Sigma-Aldrich), 0.1 M Sodium Acetate (Fisher Scientific), 38% v.v AquaPhenol (MP Biomedicals) and 5% v.v glycerol (Fisher Scientific)], and incubated at room temperature for 1 minute before centrifuging 5 minutes at 16,500 ***g***. In a new tube, the supernatant was mixed with 200 μl chloroform by vortexing 10-15 seconds. After centrifuging for 5 minutes (16,500 ***g***), the aqueous phase was transferred to new tubes. Next, 350μl 0.8M sodium citrate and 350 μl isopropanol were added, gently mixed by inverting closed tubes, and left at room temperature for 10 minutes. The samples were then centrifuged for 15 minutes at 16,500 ***g*** in a pre-cooled (4°C) centrifuge. Pellets were washed in 1 ml 70% ethanol twice, centrifuged at 16,500 ***g*** for 1 minute, before removing the ethanol, and left to air dry before being dissolved in 50 μl nuclease-free water. The amount of total RNA in each of the samples was measured by nanodrop and standardised. Samples were treated with DNase I (Promega) for 30 minutes at 37°C to remove residual DNA. First-strand cDNA synthesis was performed from 1 μg RNA, using SuperScript III Reverse Transcriptase (Invitrogen) in accordance to the supplier’s recommendations. For qPCR reactions, the Rotor-Gene SYBR Green PCR Kit (Qiagen) was used in conjunction with a Rotor-Gene Q (Qiagen) real-time PCR cycler, according to the manufacturer’s instructions. Relative gene expression was quantified using Livak’s ΔΔCT method (Livak and Schmittgen, 2001) with correction for PCR efficiency of each sample. Gene expression was normalised against average expression values of At1g13440 (GAPDH), At5g25760 (UBC) and At2g28390 (SAND family protein; Czechowski *et al.*, 2005). For biomass quantification of *Hyaloperonospora arabidopsidis* and *Plectosphaerella cucumerina* the DNA was amplify using the set of primers described in (Anderson and McDowell, 2015; Sanchez-Vallet *et al.*, 2010).

### Primer sequences.

***CMT3 SALK_148381.31.90 – genotyping:***

CMT3 LP CCCTCAACAATTAACTGACGC

CMT3 RP ATAAGAGAAGGAGCTGCTGCC

***NRPE1 - gene expression (downstream of T-DNA insertion):***

NRPE1 FW GGCGTTGACTTCATCACGGTTG

NRPE1 RV AAAGAAGCACCTGCTGTCTGAG

***ROS1 SALK_045303.23.25.x - genotyping:***

ROS1-4 LP CCAGTTAAGGACAGAACACCG

ROS1-4 RP TCGTCTTTCGATCAAATCCAC

***ROS1 SALK_045303.23.25.x – gene expression (downstream of T-DNA insertion):***

ROS1 FW CACATACGTACCCTTTCCAAAAGC

ROS1 RV GGGAATTACCTCGAAGAACG

***ROS3 SALK_022363.36.85.x - genotyping:***

ROS3 LP ATCAATGTGGCATCTAGTGGC

ROS3 RP ACCCGCCTCTTCTTCATCTAC

***At3G24900 – Microarray validation:***

At3G24900 FW.2 CACCACCAGCACAACATCCTAAAG

At3G24900 RV TACCCTATTCCCACGCCTTTCC

***At4G04500 – Microarray validation:***

At4G04500 FW TGTGTGGAAGAGGTGGATTGAAGG

At4G04500 RV TCGGAGCAGCCAAAGGATCAATG

***At4G11000 – Microarray validation:***

At4G11000 FW TGGTAACACAGTCTTCCATATCGC

At4G11000 RV ACGCAGCAATTTCATCACCTCAG

***At5G35735 – Microarray validation:***

At5G35735 FW ACAGGCTGAGGAAGAGAAACACG

At5G35735 RV.2 TGCTCCCATTGGCATTAGTACTCC

### Microarray analysis.

RNA levels were analysed using Affymetrix Arabidopsis Gene 1.0 ST arrays according to the manufacturer’s instructions. One replicate of mock treated Col-0 at 48 hrs was removed prior to data processing as it did not pass quality control. Data were RMA-normalised in R (R Core Team, 2015), using the package oligo (Carvalho and Irizarry, 2010), before analysing hybridization intensities for statistically significant differences, using the R package Limma (Ritchie *et al.*, 2015; Smyth, 2004). Raw data files and the RMA-normalised data matrix have been deposited in ArrayExpress under accession number E-MTAB-3963. The following contrasts were compared per time-point: Col-0 *Hpa*-infected versus Col-0 mock-treated, *nrpe1* mock-treated versus Col-0 mock-treated, *ros1* mock-treated versus Col-0 mock-treated, *nrpe1* *Hpa*-infected versus Col-0 *Hpa*-infected, *ros1* *Hpa*-infected versus Col-0 *Hpa*-infected, and *nrpe1* *Hpa*-infected versus *ros1* *Hpa-*infected. A difference in expression was deemed to be significant at q­-value ≤ 0.01 (method global, adjust method fdr). For heatmaps, RMA expression values were first converted to z-scores and then plotted using the heatmap.2 function from R package gplots (Warnes *et al.*, 2015).

### Analysis of sequencing data.

Bisulfite sequencing reads that are part of two published studies (Qian *et al.*, 2012; Stroud *et al.*, 2013) were downloaded from NCBI's SRA (accession numbers SRR353936-SRR353939, SRR534177, SRR534182 and SRR534193). ChIP-seq data described by Zhong *et al*. (2015) were used directly as downloaded from NCBI's GEO (series number GSE61192). Reads were pre-processed using trimmomatic (Bolger *et al.*, 2014) to remove any potential vector sequence and poor quality 3' sections in reads (sliding window width 5 quality 20), retaining only reads of which at least 36 bases remained. Data for all samples were aligned as consisting solely of single-end reads. Reads were aligned to the TAIR10 *Arabidopsis thaliana* genome sequence (Lamesch *et al.*, 2012) using Bismark v0.10.1 (Krueger and Andrews, 2011), which was set up to use Bowtie v1.0.0 (Langmead *et al.*, 2009). Alignment files were de-duplicated, using the script supplied with Bismark to remove reads that were potentially derived from the same fragment. De-duplicated alignment files were converted by using the R package methylKit, using read.bismark (Akalin *et al.*, 2012). Positions that had more than 99.9th percentile of coverage were removed from each sample. Differentially methylated positions were determined separately in three different sequence contexts: CHG, CHH and CG. Although comparisons between mutant and wild type were made separately for each study, only positions for which at least 5 reads were available in each sample across all studies were taken into account. Features were counted using BEDtools (Quinlan and Hall, 2010) and processed using perl scripts. Sequence data and genome annotations were visualised using IGV (Robinson *et al.*, 2011; Thorvaldsdottir *et al.*, 2013), of which the scalable vector graphics output was post-processed in Inkscape (Albert *et al.*, 2015). Venn diagrams were generated using BioVenn (Hulsen *et al.*, 2008).

### References

**Akalin, A., Kormaksson, M., Li, S., Garrett-Bakelman, F.E., Figueroa, M.E., Melnick, A. and Mason, C.E.** (2012) methylKit: a comprehensive R package for the analysis of genome-wide DNA methylation profiles. *Genome Biol.*, 13, R87.

**Albert, M., Andler, J.A., Bah, T., et al.** (2015) *Inkscape*, Available at: https://inkscape.org.

**Anderson, R.G., McDowell, J.M.** (2015) A PCR assay for the quantification of growth of the oomycete pathogen Hyaloperonospora arabidopsidis in Arabidopsis thaliana. *Mol Plant Pathol*, 16, 893-8.

**Bolger, A.M., Lohse, M. and Usadel, B.** (2014) Trimmomatic: a flexible trimmer for Illumina sequence data. *Bioinformatics*, 30, 2114–2120.

**Carvalho, B.S. and Irizarry, R. a.** (2010) A framework for oligonucleotide microarray preprocessing. *Bioinformatics*, 26, 2363–2367.

**Czechowski, T., Stitt, M., Altmann, T., Udvardi, M.K. and Scheible, W.-R.** (2005) Genome-Wide Identification and Testing of Superior Reference Genes for Transcript Normalization in Arabidopsis. *Plant Physiol.*, 139, 5–17.

**Hulsen, T., Vlieg, J. de and Alkema, W.** (2008) BioVenn – a web application for the comparison and visualization of biological lists using area-proportional Venn diagrams. *BMC Genomics*, 9, 488.

**Kasajima, I., Ide, Y., Ohkama-Ohtsu, N., Hayashi, H., Yoneyama, T. and Fujiwara, T.** (2012) A protocol for rapid DNA extraction from Arabidopsis thaliana for PCR analysis. *Plant Mol. Biol. Report.*, 22, 49–52.

**Krueger, F. and Andrews, S.R.** (2011) Bismark: a flexible aligner and methylation caller for Bisulfite-Seq applications. *Bioinformatics*, 27, 1571–1572.

**Lamesch, P., Berardini, T.Z., Li, D., et al.** (2012) The Arabidopsis Information Resource (TAIR): improved gene annotation and new tools. *Nucleic Acids Res.*, 40, D1202–D1210.

**Langmead, B., Trapnell, C., Pop, M. and Salzberg, S.L.** (2009) Ultrafast and memory-efficient alignment of short DNA sequences to the human genome. *Genome Biol.*, 10, R25.

**Livak, K.J. and Schmittgen, T.D.** (2001) Analysis of Relative Gene Expression Data Using Real-Time Quantitative PCR and the 2−ΔΔCT Method. *Methods*, 25, 402–408.

**Quinlan, A.R. and Hall, I.M.** (2010) BEDTools: a flexible suite of utilities for comparing genomic features. *Bioinformatics*, 26, 841–842.

**R Core Team** (2015) *R: A language and environment for statistical computing.*, Vienna, Austria: R Foundation for Statistical Computing.

**Ritchie, M.E., Phipson, B., Wu, D., Hu, Y., Law, C.W., Shi, W. and Smyth, G.K.** (2015) limma powers differential expression analyses for RNA-sequencing and microarray studies. *Nucleic Acids Res.*, 43, e47–e47.

**Robinson, J.T., Thorvaldsdóttir, H., Winckler, W., Guttman, M., Lander, E.S., Getz, G. and Mesirov, J.P.** (2011) Integrative genomics viewer. *Nat. Biotechnol.*, 29, 24–26.

**Sanchez-Vallet, A., Ramos, B., Bednarek, P., López, G., Piślewska-Bednarek, M., Schulze-Lefert, P. and Molina, A.** (2010) Tryptophan-derived secondary metabolites in Arabidopsis thaliana confer non-host resistance to necrotrophic Plectosphaerella cucumerina fungi. *Plant J*. 63, 115-27.

**Smyth, G.K.** (2004) Linear Models and Empirical Bayes Methods for Assessing Differential Expression in Microarray Experiments Linear Models and Empirical Bayes Methods for Assessing Differential Expression in Microarray Experiments. *Stat. Appl. Genet. Mol. Biol.*, 3, 1–26.

**Thorvaldsdottir, H., Robinson, J.T. and Mesirov, J.P.** (2013) Integrative Genomics Viewer (IGV): high-performance genomics data visualization and exploration. *Brief. Bioinform.*, 14, 178–192.

**Warnes, G.R., Bolker, B., Bonebakker, L., et al.** (2015) *gplots: Various R Programming Tools for Plotting Data*, Available at: http://cran.r-project.org/web/packages/gplots/index.html.

**Zhong, X., Hale, C.J., Nguyen, M., et al.** (2015) DOMAINS REARRANGED METHYLTRANSFERASE3 controls DNA methylation and regulates RNA polymerase V transcript abundance in *Arabidopsis*. *Proc. Natl. Acad. Sci.*, 112, 911–916.
