# Supplementary material for: The role of DNA (de)methylation in immune responsiveness of Arabidopsis
Source: Plant J. 2016 Sep 7;88(3):361–74. doi: 10.1111/tpj.13252 (PMC5132069; doi:10.1111/tpj.13252)
Supplement: Supplementary file 10 [file TPJ-88-361-s010.docx]

**Supporting Information Legends**

**Figure S1:** Genetic characterization of selected mutants. (a) Genotyping of *cmt3*, *ros1* and *ros3* mutants. (b) Expression levels of *ROS1* (left) and *NRPE1* (right) in the *ros1* and *nrpe1* mutant, respectively, relative to wild-type Col-0. Primers usedin (a) and (b) are described in Methods S1.

**Figure S2:** Repeats of pathogenicity assays to determine basal resistance in DNA (de)methylation mutants against *Hyaloperonospora arabidopsidis* (*Hpa*). (a) Trypan blue visual scoring of *Hpa* infections, as in Figure 1a. (b) RT-qPCR-based quantification of the presence of *Hpa* DNA relative to Arabidopsis DNA. Infected material harvested three days post-inoculation of Col-0 was taken as reference.

**Figure S3:** Basal resistance phenotypes of Col-0, *nrpe1* and *ros1* to the necrotrophic fungi *Plectosphaerella cucumerina* and *Alternaria brassicicola*. (a) Representative images of *P. cucumerina* lesion formation on Col-0, *nrpe1* and *ros1*. Quantification of diameters is presented in Figure 3a. (b) RT-qPCR-based estimation of the presence of *P. cucumerina* DNA relative to Arabidopsis DNA. Values are presented with Col-0 four days post-inoculation (dpi) as reference. (c) Susceptibility of *ros1* and Col-0 to *Alternaria brassicicola*. Plants were drop-inoculated at five weeks post germination with 6 μl droplets of a 10^6^ spores ml^-1^ spore suspension. At 14 days post inoculation, disease symptoms were assessed in a total of 40 leaves from 10 plants after trypan blue staining. Classes are defined as follows; lowest: no hyphal colonisation of the leaf – arrow indicates spore; intermediate: low/intermediate density of hyphal colonisation – arrow indicates hypha; highest: dense hyphal colonisation with a presence of necrotic lesions. The asterisk indicates a statistically significant difference in distribution of classes (*χ*^2^ test, *p* < 0.05). Scale bars: 100 μm. *A. brassicicola* spores were prepared as described for *P. cucumerina* except *A. brassicicola* was grown on PDA-CaCO_3_ plates.

**Figure S4:** Transcript levels of 166 *Hpa-*inducible genes with augmented induction in *nrpe1* and/or repressed induction in *ros1*. Genes were selected when differentially expressed between *ros1* and *nrpe1*, as well as between Col-0 and *ros1*, and/or between Col-0 and *nrpe1*, at either 48 or 72 hours post-inoculation (hpi). Heat map projections represent Z-scorse of transcript levels.

**Figure S5:** Micro-array validation of transcriptional profiles from an independent *Hpa* experiment. Heat maps visualise the z-scores derived from the RMA-normalised expression values from the array and the -ΔCt values from RT-qPCR experiments. Bar graphs show expression relative to mockinoculated Col-0 at 48 hours post inoculation (hpi).

**Figure S6:** Schematic overview of the 2 kb defence gene promoters that contain one or more TEs, have three or more hypermethylated cytosines in the *ros1* mutant, and/or have three or more hypo-methylated cytosines in the *nrpe1* mutant. Cytosine methylation is indicated by vertical ticks/lines. Grey vertical lines: cytosines for which ≥20% of the reads indicate methylation (i.e. C to T conversion after bisulfite treatment); Blue ticks: cytosines that are hypo-methylated in *nrpe1*; Red ticks: cytosines that are hypermethylated in *ros1*. Green horizontal bars: TEs. Purple bars: other gene models that fall into the 2 kb region upstream from the transcriptional start site of the gene. Teal histograms indicate association with NRPE1 as deternined by ChIP-seq (Zhong et al., 2015); the superimposed darker colour indicates background levels (ChIP-seq data obtained from the *nrpe1-12* mutant).

### Table S1: Annotations of 25 candidate defence-regulatory genes that are *cis*-regulated by NRPE1- and/or ROS1-dependent DNA (de-)methylation.

### Supplemental methods file: Details about plant growth conditions, basal resistance assays, staining procedures & resistance classifications, nucleic acid extractions & qPCR, primer sequences, microarray analysis, and analysis of sequencing data.

### Supplemental data file 1: Gene transcripts showing statistically significant differences in normalized hybridization signal (Affymetrix Arabidopsis Gene 1.0 ST arrays) between Col‑0, *nrpe1*, and *ros1* at 48 and 72 hours after mock or *Hpa* inoculation*.*
